# Supporting activities of cognate redox partners for sterol-metabolizing P450 enzymes in *Mycobacterium neoaurum*

**DOI:** 10.1016/j.jbc.2026.113116

**Published:** 2026-05-07

**Authors:** Yunjie Liu, Yue Zhao, Weihan Sun, Nian Li, Yunjun Pan, Li Ma, Shengying Li

**Affiliations:** 1 State Key Laboratory of Microbial Technology, Shandong University, Qingdao, Shandong, China; 2 Melton International School, Qingdao, Shandong, China; 3 Laboratory for Marine Biology and Biotechnology, Qingdao Marine Science and Technology Center, Qingdao, Shandong, China

**Keywords:** steroid, *Mycobacterium neoaurum*, cytochrome P450 enzymes, redox partner, ferredoxin, ferredoxin reductase

## Abstract

Steroids with anti-inflammatory, anti-allergic, endocrine-regulating, and other pharmaceutical activities represent the second most widely used class of drugs worldwide, following antibiotics. Their industrial production primarily relies on *Mycobacteria*-mediated biotransformation of sterols into key intermediates, followed by chemical or enzymatic modifications. While the sterol metabolic pathways in *Mycobacteria* have been intensively studied, the identification and functional characterization of key enzymes, particularly cytochrome P450 enzymes (CYPs or P450s) and their cognate redox partners, remain incomplete. Here, we heterologously expressed 24 P450s, 10 ferredoxin reductases (FdRs), and 12 ferredoxins (Fdxs) from *Mycobacterium neoaurum* ZC-1 in *Escherichia coli*. *In vitro* biochemical experiments identified five P450 enzymes (CYP125A76, CYP125A77, CYP125A78, CYP142A12, and CYP124A1) capable of catalyzing sterol side-chain terminal oxidation. Screening 120 redox partner combinations revealed FdR4662/Fdx4443 as the optimal cognate redox partners for all five P450 enzymes. With this redox partner pair, CYP142A12 achieved a conversion ratio of 89% for 4-cholesten-3-one with NADH as the preferred cofactor. Structural analyses indicate that the electron-transfer efficiency is primarily governed by electrostatic complementarity around the Fe–S cluster, the redox-center distance between the Fe–S cluster and heme-iron, and the FAD-to-cluster distance within the FdR–Fdx complex. These findings highlight the critical role of redox partner selection in enhancing P450 catalytic efficiency and provide a solid foundation for engineering high-efficiency industrial strains to improve steroid biomanufacturing and reduce production costs.

Steroids play a central role in the treatment of diverse medical conditions including inflammation, allergies, cardiovascular diseases, endocrine disorders, and cancer (1, 2). Globally, steroid drug production exceeds one million tonnes per year, with sales estimated at approximately $10 billion in 2018 (2, 3, 4). Traditional steroid synthesis relies on the semisynthetic conversions of diosgenin, a steroidal sapogenin primarily extracted from the tubers of yam (*Dioscorea* spp.). This process comprises two primary stages: the preparation of the key intermediate pregnadienolone acetate, followed by its chemical modifications to yield various steroid pharmaceuticals (5, 6). However, challenges such as the fluctuation in yam prices and negative environmental impacts of the synthetic process have hindered its sustainable industrial application (3, 7, 8, 9). To address these issues, the pharmaceutical industry has been exploring more cost-effective and sustainable alternatives, including use of other raw materials such as by-products of vegetable oil processing and paper mill wastewater, both of which are rich in phytosterols (10). These sterol mixtures can be bio-transformed by specific microorganisms, such as *Mycobacterium neoaurum*, yielding key steroid intermediates including androstenedione (AD), androsta-1,4-diene-3,17-dione, and 9-hydroxyandrost-4-ene-3,17-dione (11, 12, 13).

The biotransformation of phytosterols to AD consists of two key stages: the initial oxidation of the steroid nucleus and subsequent degradation of the side chain. Oxidation at the steroid nucleus typically initiates the process catalyzed by enzymes such as cholesterol oxidase or 3*β*-hydroxysteroid dehydrogenase, yielding 4-cholesten-3-one (14, 15, 16, 17). Subsequently, the intermediate undergoes C26 hydroxylation and further oxidative modifications, resulting in the formation of steroidal C26-acids (18, 19, 20, 21, 22, 23, 24). This degradation process employs fatty acid *β*-oxidation and is mediated by a cascade of nine catabolic enzymes integral to the 14-step process that systematically cleaves the sterol side chain into simpler molecules (25, 26, 27, 28). Upon complete degradation of the side chain, sterols are converted into AD. This transformation hinges on C26 hydroxylation, a critical step within the entire degradation pathway, which is catalyzed by cytochrome P450 enzymes, including CYP125, CYP142, and CYP124 family members (Fig. S1).

P450 enzymes exhibit remarkable functional diversity, contributing to a wide range of biological processes, including drug metabolism, natural product biosynthesis, xenobiotic degradation, steroid biogenesis, and drug target exploration (29, 30, 31). The canonical P450 catalytic cycle involves the sequential delivery of two electrons from NAD(P)H to the heme iron with the assistance of redox partner proteins, thereby enabling the activation of molecular oxygen (32, 33, 34, 35, 36). In bacterial class I P450 systems, the electron transfer is typically mediated by two redox partner proteins: an FAD-containing ferredoxin reductase (FdR) and a small iron-sulfur–bearing ferredoxin (Fdx) (37, 38). The efficiency of the FdR→Fdx→P450 electron transport chain significantly influences the catalytic rate (39, 40, 41). A bacterial genome often encodes multiple Fdxs and FdRs (42, 43, 44), which are typically dispersed and located far away from P450 genes (45, 46). This lack of proximity and genetic association underscores the challenge of determining the native optimal redox partners for bacterial P450 enzymes, leaving this area of study largely untapped (37, 47, 48, 49, 50).

Cytochrome P450 (CYP) enzymes from the industrial strain *M*. *neoaurum* ZC-1 play a crucial role in sterol metabolism (1). However, systematic research on these enzymes, particularly their cognate redox partners, remains underexplored. Here, we conducted a comprehensive series of *in vitro* enzyme assays on 24 P450 enzymes, identifying five that are directly involved in sterol metabolism. Further biochemical characterization of 10 FdRs and 12 Fdxs from the ZC-1 strain revealed their cofactor specificity and inherent electron transfer capabilities. Structural analysis provided deeper insight into the P450–Fdx and Fdx–FdR interactions, which are key determinants of P450 activity. This work represents the first systematic screening and optimization of *M*. *neoaurum* redox partner combinations. The results demonstrate that FdR4662/Fdx4443 is the optimal cognate redox partners for the five sterol-metabolizing P450 enzymes. We expect that these findings will provide both mechanistic insights and practical guidance for the development and optimization of high-yield industrial strains, thereby advancing the application of *M. neoaurum* in steroid biomanufacturing.

## Results

### Acquisition of cytochrome P450, ferredoxin, and ferredoxin reductase genes

**Figure 1:**
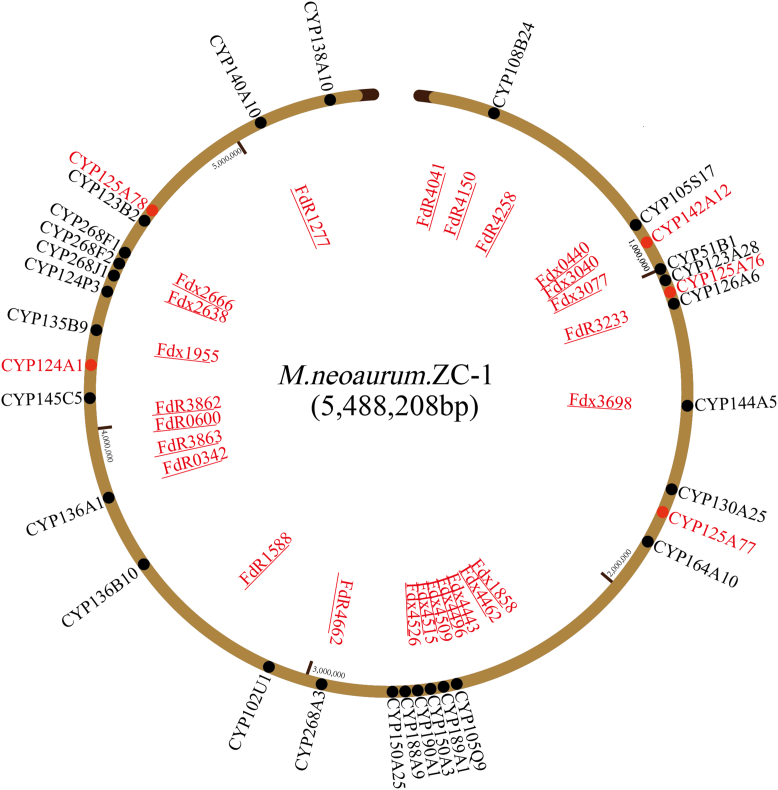
**Genome maps of *Mycobacterium neoaurum* ZC-1.** The locations of the studied 12 Fdxs, 10 FdRs, and 5 P450s genes are marked with *red lines* and *red dots*, respectively.

The genome of *M*. *neoaurum* strain ZC-1 was sequenced using second generation high-throughput sequencing technology. Through careful analysis and annotation, a total of 32 P450, 11 FdR, and 14 Fdx genes were identified (Fig. 1 and Table S1). The 32 P450 enzymes were classified into 22 P450 families (with > 40% amino acid sequence identity) by Prof. David R. Nelson (51).

### Heterologous expression and purification of cytochrome P450s, ferredoxins, and ferredoxin reductases

*Escherichia coli* BL21(DE3) was utilized for heterologous expression of all P450 enzymes, Fdxs, and FdRs of *M*. *neoaurum* ZC-1. As a result, only three His-tagged recombinant P450 enzymes (CYP105S17, CYP268F2, and CYP125A78) were straightforwardly expressed in soluble form and purified *via* Ni-NTA affinity chromatography. For the remaining 29 P450 enzymes, 14 Fdxs and 11 FdRs that were initially insoluble, different strategies such as codon optimization and *N*-terminal fusion of maltose-binding protein were applied. These efforts eventually led to soluble expression of 21 P450s, 12 Fdxs, and 10 FdRs (Figs. S2 and S3 and Table S2). However, eight P450 enzymes (CYP102U1, CYP124P3, CYP136A1, CYP138A10, CYP135B9, CYP136B10, CYP145C5, and CYP188A9), two Fdxs (Fdx0440 and Fdx2638), and one FdR (FdR1588) remained insoluble, despite multiple strategies including alteration of induction conditions, addition of solubility tags, and protein denaturation-renaturation.

### Functional characterization of cytochrome P450 enzymes from *M. neoaurum* ZC-1

Functional expression of 24 P450 enzymes was confirmed by analyzing their CO•Fe(II) *versus* Fe(II) difference spectra. Among them, 22 P450 enzymes displayed the characteristic absorption peak at 450 nm (Fig. S4), consistent with the typical spectral properties of functionally active P450 enzymes (52). However, CYP140A10 and CYP123A28 did not show the 450 nm peak and instead displayed an absorption peak at 420 nm (Fig. S4), indicative of improper folding in *E. coli* or P420 formation (53, 54).

**Figure 2:**
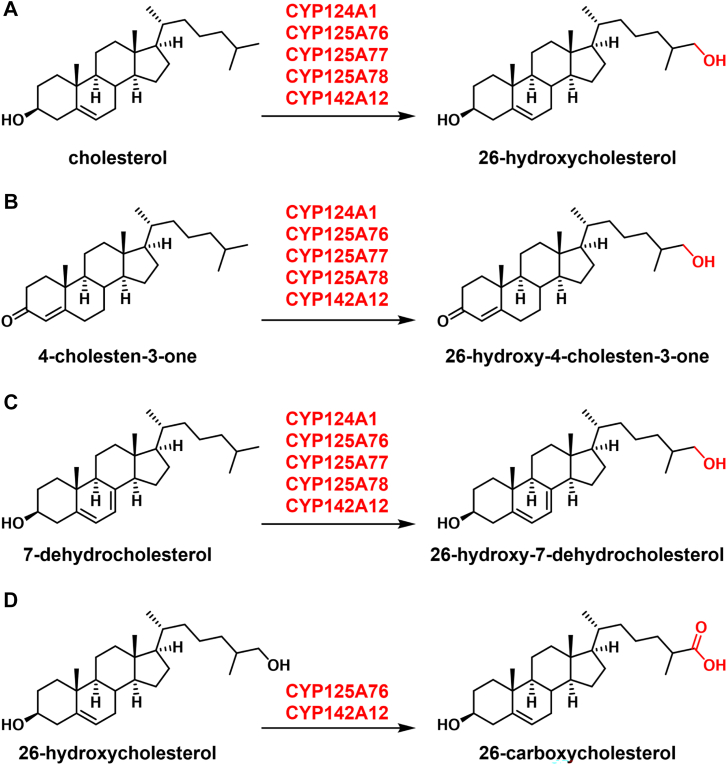
**Reactions catalyzed by *Mycobacterium neoaurum* P450 enzymes.***A*, C26 hydroxylation of cholesterol to 26-hydroxycholesterol. *B*, C26 hydroxylation of 4-cholesten-3-one to 26-hydroxy-4-cholesten-3-one. *C*, C26 hydroxylation of 7-dehydrocholesterol to 26-hydroxy-7-dehydrocholesterol. *D*, Further oxidation of 26-hydroxycholesterol to 26-carboxycholesterol. The P450 enzymes responsible for each reaction are indicated above the arrows, and the newly introduced functional groups are highlighted in red.

To evaluate the catalytic competence of the 24 soluble P450 enzymes toward steroidal substrates, *in vitro* single-substrate, multi-enzyme reactions were performed using a diverse set of steroidal compounds including cholesterol, 4-cholesten-3-one, 7-dehydrocholesterol, 3-*β*-hydroxy-5,24-cholestadiene, lanosterol, 24,25-dihydrolanosterol, episterol, vitamin D3, androstenedione, testosterone, progesterone, and estradiol (55, 56, 57, 58, 59) (Fig. S5). Since the Fe_2_S_2_ ferredoxin *Sel*Fdx1499 and the plastidic-type ferredoxin reductase *Sel*FdR0978 from *Synechococcus elongatus* PCC7942 were reported to exhibit high electron transfer efficiency and broad compatibility with class I P450 enzymes (48), we used these redox partners to reconstitute the *in vitro* P450 reactions. Experimentally, each 6-h reaction at 30 °C contained one steroid substrate, five P450 enzymes, *Sel*FdR0978/*Sel*Fdx1499, and NADPH. Gas chromatography (GC) or high-performance liquid chromatography (HPLC) analyses revealed product formation in the reactions containing CYP125A76, CYP125A77, CYP125A78, CYP142A12, and CYP124A1, which catalyzed the oxidation of cholesterol, 4-cholesten-3-one, and 7-dehydrocholesterol. Among these, 4-cholesten-3-one is a known substrate for CYP125 family enzymes (60). No products were detected for other substrates. These results suggest that sterol metabolism in *M*. *neoaurum* ZC-1 strain should primarily involve CYP125A76, CYP125A77, CYP125A78, CYP142A12, and CYP124A1 (Fig. 2). These enzymes catalyze the oxidation of sterol terminal C-H bond before CoA ligation, consistent with previous reports (61).

### Ferredoxin reductase activity and cofactor preference

**Figure 3:**
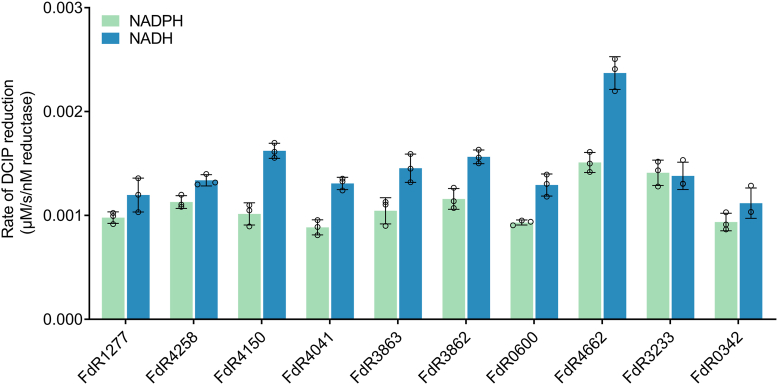
**DCIP reduction activities of eleven FdRs when using NADH or NADPH as electron donor.** Results are expressed as mean ± SD. Individual data points (n = 3 replicates) are shown as *black circles*.

We measured the FdR activity using 2,6-dichloroindophenol (DCIP) as an artificial electron acceptor. The cofactor preference was evaluated by comparing the DCIP-reducing activities of FdR supported by NADPH and NADH. All 11 FdRs were capable of using both NADH and NADPH, but showed a clear preference for NADH over NADPH as a cofactor (Fig. 3). Consistent with these results, protein sequence analysis (Fig. S6) revealed the presence of NADH-preference motifs. Specifically, eight out of the eleven FdRs harbor the conserved NADH-binding motif “GXTX,” instead of the canonical NADPH-binding motif “GXAX” (62). Within this group, three FdRs (FdR1277, FdR4150, and FdR4041) harbor the “GSGITP” consensus sequence, while two (FdR0600 and FdR4662) carry the “GIGITP” motif. The remaining three (FdR4258, FdR3863, and FdR0342) have the sequences “RMGHTD,” “DGITD,” and “ALGRTD,” respectively, all consistent with the “GXTX” consensus. Of the three FdRs lacking the “GXTX” motif, one (FdR3862) contains a nonrecognizable motif, whereas the other two carry the sequences “EQLGTE” (FdR1588) and “QLERTD” (FdR3233), both retaining the conserved threonine residue at the fifth position.

Further analysis showed that FdR4662, when paired with its optimal electron donor NADH, displayed the highest DCIP reduction activity at (2.4 ± 0.2) × 10^−^^3^ μM s^−1^ nM^−1^. Following this, FdR4150, FdR3862, and FdR3863 showed reduction rates of (1.6 ± 0.1) × 10^−3^, (1.6 ± 0.1) × 10^−3^, and (1.5 ± 0.5) × 10^−3^ μM s^−1^ nM^−1^, respectively, with NADH. In contrast, FdR1277 and FdR0342 showed the lowest DCIP reduction rate, with values of (1.2 ± 0.2) × 10^−3^ and (1.1 ± 0.2) × 10^−3^ μM s^−1^ nM^−1^, respectively.

**Figure 4:**
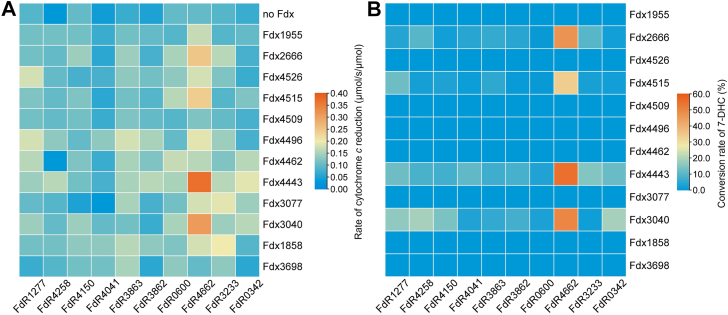
**Comparisons of the activities of 120 distinct redox partner pairs.***A*, electron transfer rate for cyt *c* reduction by different redox partner combinations (10 FdRs and 12Fdxs). The shading colors reflect the variation in cyt *c* reduction rates. *B*, conversion ratios of 7-dehydrocholesterol by CYP125A77 when supported by 120 different redox partners pairs (10 FdRs × 12 Fdxs; [Fdx] = 10 μM). The shading colors indicate the substrate conversion ratios. Detailed quantitative data, including mean values and standard deviations (SD) from three independent biological replicates, are provided in Tables S5 and S6.

The electron transport chain of NAD(P)H→FdR→Fdx→heme directly influences P450 catalysis. It is essential to evaluate its efficiency for optimizing P450 catalytic system. Thus, we constructed an electron transport chain with NADH, FdR, Fdx, and cytochrome *c* (cyt *c*) as a surrogate electron acceptor to evaluate the *in vitro* electron transfer efficiency of 120 combinations of redox partners (10 FdRs and 12 Fdxs) from strain ZC-1. As a result, all combinations efficiently reduced cyt *c*. Notably, in the absence of Fdx, all 10 FdRs could directly transfer electrons to cyt *c* at the rates ranging from 0.03 to 0.10 μM s^-1^ μM^−1^ (Fig. 4*A*), consistent with our previous findings (48). Among all combinations, four pairs of redox partners exhibited relatively higher cyt *c* reduction rates, exceeding 0.20 μM s^−1^ μM^−1^. The most efficient combinations included FdR4662 paired with Fdx2666 (0.25 μM s^−1^ μM^−1^), Fdx4515 (0.24 μM s^−1^ μM^−1^), Fdx4443 (0.37 μM s^−1^ μM^−1^), and Fdx3040 (0. 31 μM s^−1^ μM^−1^) (Fig. 4*A*). These results revealed differences in electron transfer efficiency among various FdR and Fdx combinations, highlighting the potential to enhance P450 enzymatic activities by selecting appropriate FdR-Fdx pairings.

### Catalytic activities of CYP125A77 when supported by different redox partner combinations

Sequence analysis using T-COFFEE software and ESPript 3.0 (63) showed that CYP125A77 shares 53% and 69% sequence identity with CYP125A76 and CYP125A78, respectively, while exhibiting lower similarity to CYP142A12 and CYP124A1 (Fig. S7). Using CYP125A77 as a model, we evaluated the impact of redox partner combinations on P450 catalytic efficiency. A reaction network of 120 redox partner pairings was generated by combining 10 FdRs with 12 Fdxs. The results showed that CYP125A77 was able to catalyze the hydroxylation of 7-dehydrocholesterol to yield 26-hydroxy-7-dehydrocholesterol (Fig. 2*C*). The substrate conversions varied significantly depending on the pairing of redox partners (Fig. 4*B*). Among the combinations tested, FdR4662 pairing with Fdx4443, Fdx2666, Fdx3040, and Fdx4515 led to high catalytic activities of CYP125A77, with substrate conversion ratios of 55.3 ± 1.6%, 47.3 ± 2.5%, 49.7 ± 3.1%, 34.0 ± 2.5%, respectively, while the conversion ratios for the rest combinations were below 30% (Fig. 4*B*).

Fdxs are classified by their iron-sulfur cluster composition into Fe_2_S_2_-, Fe_3_S_4_-, and Fe_7_S_8_-types (Fig. S8 and Table S3) (50, 64). Activity analysis showed that the Fe_3_S_4_-type Fdxs including Fdx4443, Fdx2666, Fdx4515, and Fdx3040, well supported the CYP125A77-catalyzed oxidation of 7-dehydrocholesterol in combination with 10 different FdRs. Notably, the Fdx4443/FdR4662 pair achieved the highest substrate conversion ratio. In contrast, several Fdxs, including Fdx1955, Fdx4526, Fdx4509, Fdx4496, Fdx4462, Fdx3077, Fdx1858, and Fdx3698 failed to support the CYP125A77-catalyzed reaction with 7-dehydrocholesterol when paired by any FdR. These findings underscore the importance of specific Fdx–P450 interactions in enabling effective catalysis (37).

### The P450 activities supported by different redox partner combinations

In the previous section, four highly efficient pairs were identified. These results correlated well with the electron transfer efficiency determined by using cyt *c* as the terminal electron acceptor. Building on these findings, we further evaluated the outperforming redox partner pairs for CYP125A76, CYP125A78, CYP142A12, and CYP124A1. A reaction matrix comprising 60 P450 assays was constructed by pairing the five P450 enzymes with the four redox partner combinations (*i.e.*, FdR4662/Fdx2666, FdR4662/Fdx4515, FdR4662/Fdx4443, and FdR4662/Fdx3040). Each combination was tested with three sterol substrates: cholesterol, 4-cholesten-3-one, and 7-dehydrocholesterol. Among them, 4-cholesten-3-one is a known natural substrate for CYP125 enzymes. The structural variety of the sterol substrates, distinguished by different substitution patterns on specific cyclic moieties, enabled evaluation of the substrate selectivity of these P450 enzymes.

**Figure 5:**
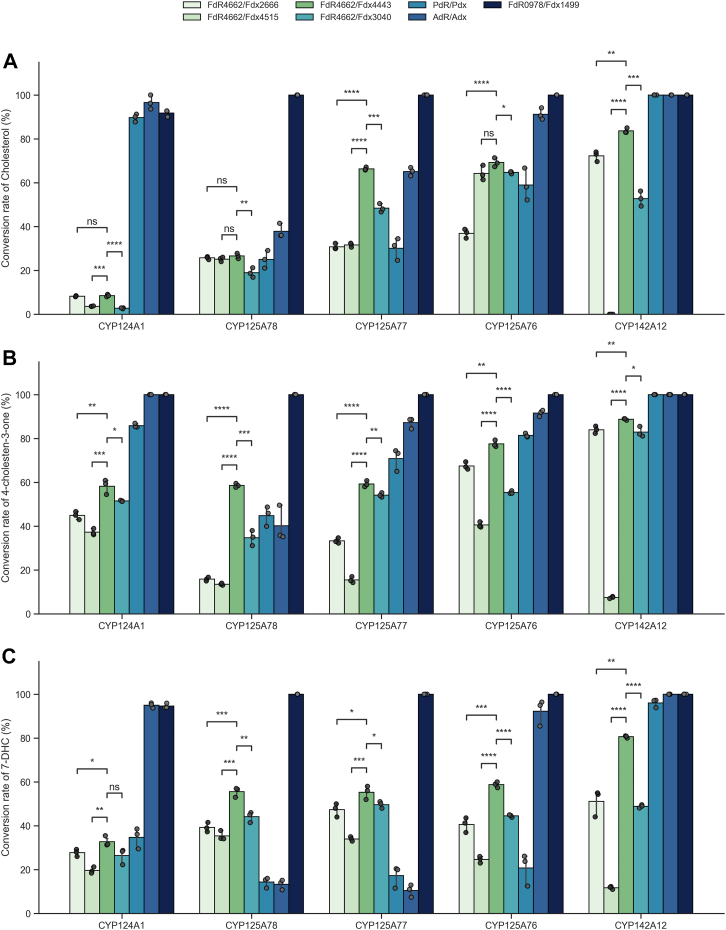
**The supporting activities of cognate and heterologous redox partners for P450 enzymes.** Conversion ratios of cholesterol (*A*), 4-cholesten-3-one (*B*), and 7-DHC (*C*) by five P450 enzymes when paired with four cognate redox partner combinations and three pairs of heterologous redox partners. Statistical significance was evaluated using the Student’s *t* test (∗*p* < 0.05, ∗∗*p* < 0.01, ∗∗∗*p* < 0.001, ∗∗∗∗*p* < 0.0001, ns: *p* > 0.05, not significant). Data are presented as mean ± SD, with *black dots* representing individual measurements (n = 3).

All reaction products were analyzed using HPLC or GC analysis (Figs. S9–S16). Specifically, when cholesterol served as the substrate, CYP125A76, CYP125A77, CYP125A78, CYP142A12, and CYP124A1 catalyzed its conversion to 26-hydroxycholesterol (Figs. 2*A* and S9). Among the four tested *M. neoaurum* cognate redox partner pairs, FdR4662/Fdx4443 exhibited the highest efficiency, supporting optimal cholesterol-hydroxylating activity for CYP142A12, CYP125A76, CYP125A77, CYP125A78, and CYP124A1, with conversion ratios of 83.7 ± 1.2%, 69.2 ± 2.0%, 66.3 ± 0.7%, 26.6 ± 1.2%, and 8.5 ± 0.5%, respectively (Fig. 5*A*). FdR4662/Fdx3040 gave conversion ratios of 52.8 ± 3.4%, 64.7 ± 0.6%, 48.4 ± 1.9%, and 18.9 ± 2.2% for CYP142A12, CYP125A76, CYP125A77, and CYP125A78, respectively. FdR4662/Fdx2666 supported CYP142A12 (72.2 ± 2.3%), CYP125A76 (36.9 ± 2.1%), and CYP125A77 (30.8 ± 1.4%). Interestingly, FdR4662/Fdx4515 failed to support the activity of CYP142A12, whereas it enabled the conversion ratios of 64.3 ± 3.4% and 31.6 ± 0.9% for CYP125A76 and CYP125A77, respectively. CYP125A76 in combination with FdR4662/Fdx4443 yielded a trace amount of 26-aldehyde cholesterol (Figs. S9 and S14), which likely resulted from over-oxidation of 26-hydroxycholesterol. Additionally, CYP142A12 catalyzed the conversion of 26-hydroxycholesterol to 26-carboxycholesterol (Figs. 2*D* and S10), consistent with the previous findings on sterol side-chain oxidation in *M. neoaurum* (65, 66).

When 4-cholesten-3-one was used as the substrate, all five P450 enzymes converted it into 26-hydroxy-4-cholesten-3-one (Figs. 2*B* and S15). Among the four pairs of cognate redox partners, FdR4662/Fdx4443 again showed the highest activity, supporting the catalytic activity of CYP142A12 (88.8 ± 1.6%), CYP125A76 (77.5 ± 1.6%), CYP125A77 (59.3 ± 1.4%), CYP125A78 (58.6 ± 0.4%), and CYP124A1 (58.2 ± 3.4%) towards 4-cholesten-3-one (Fig. 5*B*). FdR4662/Fdx3040 supported CYP142A12 (82.9 ± 2.3%), CYP125A76 (55.4 ± 0.7%), CYP125A77 (54.2 ± 1.0%), and CYP125A78 (34.7 ± 3.5%). FdR4662/Fdx2666 led to the conversion ratios of 84.0 ± 1.7%, 67.5 ± 1.7%, 33.3 ± 1.2%, and 15.9 ± 0.9% for CYP142A12, CYP125A76, CYP125A77, and CYP125A78, respectively. In contrast, FdR4662/Fdx4515 exhibited the lowest activity, with the corresponding conversion ratios of 7.5 ± 0.5%, 40.5 ± 1.3%, 15.5 ± 1.4%, 13.5 ± 0.5%, and 37.2 ± 1.5% for CYP142A12, CYP125A76, CYP125A77, CYP125A78, and CYP124A1, respectively. Notably, CYP142A12 gave >80% conversions with FdR4662/Fdx3040 and FdR4662/Fdx2666.

We further evaluated the conversion of 7-dehydrocholesterol to 26-hydroxy-7-dehydrocholesterol by the five P450 enzymes (Figs. 2*C* and S12). Again, among the four *M. neoaurum* cognate redox partner pairs, FdR4662/Fdx4443 exhibited the highest activity (Fig. 5*C*). In contrast, FdR4662/Fdx4515 demonstrated the lowest activity, with conversion ratios of 11.8 ± 0.7%, 24.7 ± 1.5%, 34.0 ± 1.0%, 35.4 ± 2.1%, and 19.6 ± 1.5% for CYP142A12, CYP125A76, CYP125A77, CYP125A78, and CYP124A1, respectively. Medium activities were observed for FdR4662/Fdx3040 and FdR4662/Fdx2666 pairs, giving conversion ratios between 39 to 51% for most P450 enzymes (Fig. 5*C*).

The selection of appropriate redox partners is critical for reconstituting the optimal P450 enzyme activity. For instance, CYP109D1 from *Sorangium cellulosum* exhibited a 2- to 3-fold activity enhancement with *Bt*Adx/*Bt*AdR (*Bt* stands for *Bos taurus*) when compared to its native redox partners (67). In our previous study, we compared three pairs of frequently used surrogate redox partners including *Sel*Fdx1499/*Sel*FdR0978, Adx/AdR, and Pdx/PdR in terms of their electron transfer properties. The results showed that *Sel*Fdx1499/*Sel*FdR0978 are almost always the best redox partners for a select number of P450s (48, 50). In this study, we again screened these three pairs of redox partners in combination with five P450 enzymes, thus making 45 reactions towards three substrates (Figs. 5, S17, and S18).

When using *Sel*Fdx1499/*Sel*FdR0978, all enzymes except CYP124A1 achieved 100% cholesterol conversion. CYP125A76 and CYP142A12 primarily produced 26-carboxycholesterol, whereas CYP125A77 and CYP125A78 predominantly generated 26-hydroxycholesterol. When coupled with Adx/AdR, CYP142A12, CYP124A1, and CYP125A76 showed high substrate consumption ratios of 100 ± 0%, 96.6 ± 3.2%, and 91.2 ± 2.7%, respectively, while CYP125A77 and CYP125A78 exhibited lower activities (65.1 ± 1.9% and 37.8 ± 3.2% conversions). The Pdx/PdR pair supported 100 ± 0% and 89.7 ± 1.8% cholesterol conversions for CYP142A12 and CYP124A1, respectively, but showed reduced performance with other P450 enzymes.

For 4-cholesten-3-one, CYP142A12 achieved complete substrate consumption with all heterologous redox partners. CYP124A1 gave complete conversion with both *Sel*Fdx1499/*Sel*FdR0978 and Adx/AdR, but only 85.8 ± 0.9% with Pdx/PdR. CYP125A76 showed conversion ratios of 100 ± 0%, 91.6 ± 1.4%, and 81.4 ± 0.9% with *Sel*Fdx1499/*Sel*FdR0978, Adx/AdR, and Pdx/PdR, respectively. CYP125A77 and CYP125A78 showed the best performance with *Sel*Fdx1499/*Sel*FdR0978, while CYP125A78 exhibited significant decreases in substrate conversion when paired with other two systems. In the case of 7-dehydrocholesterol, *Sel*Fdx1499/*Sel*FdR0978 enabled complete conversion for CYP125A76, CYP125A77, and CYP125A78. CYP125A76 also reached 92.3 ± 5.9% conversion with Adx/AdR, while CYP125A77 and CYP125A78 showed lower activities with *Sel*Fdx1499/*Sel*FdR0978. CYP124A1 achieved approximately 95% conversion with both *Sel*Fdx1499/*Sel*FdR0978 and Adx/AdR, while CYP142A12 achieved complete conversion with both redox systems. Notably, CYP142A12 and CYP125A76 fully converted 7-dehydrocholesterol into 26-carboxy-7-dehydrocholesterol with *Sel*Fdx1499/*Sel*FdR0978, while CYP125A77 and CYP125A78 mainly produced 26-hydroxy-7-dehydrocholesterol (Fig. S12). Taken together, these results indicate that *Sel*Fdx1499/*Sel*FdR0978 are still the most promising redox partners, outperforming even the native redox systems (Fig. 5).

### Determination of substrate-binding affinities of five P450 enzymes

To evaluate the binding affinities of five P450 enzymes toward sterol substrates, the dissociation constant (*K*_*D*_) values for cholesterol, 4-cholesten-3-one, and 7-dehydrocholesterol were determined using UV-visible absorption titrations (68) (Table 1 and Fig. S19). The results showed that CYP125A76 exhibited the highest affinity for cholesterol, which was approximately threefold higher than that of CYP142A12 and fivefold higher than that of CYP125A77. Consistently, CYP125A76 also displayed the strongest affinity for 4-cholesten-3-one compared to CYP142A12, CYP125A77, and CYP124A1. Again, CYP125A76 showed a markedly higher affinity for 7-dehydrocholesterol, approximately 8- to 9-fold greater than that of other enzymes examined. The detailed *K*_*D*_ values for all enzyme–substrate complexes are summarized in Table 1. These results correlate with the enzymatic activity data, demonstrating that CYP125A76 and CYP142A12 exhibit higher catalytic efficiency at least partially due to their stronger substrate binding, while the lower substrate-binding affinities of CYP125A78 and CYP124A1 are linked to their reduced catalytic efficiency. Of note, CYP124A1 exhibited a type II inhibitor-like spectral shift with cholesterol and 7-dehydrocholesterol, but the *K*_*D*_ values could not be precisely determined. These type II–binding spectra (Fig. S19) likely reflect weak coordination between the substrate and the P450 heme, possibly due to suboptimal substrate orientation or binding at more distal positions within the P450 active site. Similarly, accurate *K*_*D*_ measurement for CYP125A78 was not possible. Furthermore, considering the presence of hydroxyl groups in cholesterol and 7-dehydrocholesterol, the observed spectral changes may also be interpreted as reverse type I binding. In this case, the hydroxyl group of the ligand might stabilize the heme-bound water molecule or alternatively displace it and directly coordinate with the heme iron.

**Table 1:** Substrate-binding affinities of sterol-oxidizing P450 enzymes from *Mycobacterium neoaurum* ZC-1

Cytochrome P450s	Cholesterol	4-Cholesten-3-one	7-Dehydrocholesterol
	*K*_*D*_ (μM)		
CYP125A76	0.2 ± 0.0	0.5 ± 0.1	0.2 ± 0.0
CYP125A77	0.9 ± 0.1	2.2 ± 0.1	1.3 ± 0.2
CYP125A78	N.D.	N.D.	N.D.
CYP142A12	0.6 ± 0.1	0.9 ± 0.2	1.5 ± 0.3
CYP124A1	N.D.	4.2 ± 0.5	N.D.

N.D., not determined.

### Mechanistic insights into redox partner support of high P450 catalytic activity

To gain deeper mechanistic insights into the higher supporting activity of specific redox partner pairs, we employed AlphaFold3 to predict the three-dimensional structures of CYP142A12 and six ferredoxins, including five high-efficiency partners (*Sel*Fdx1499, Fdx4443, Fdx2666, Fdx3040, and Fdx4515) and one low-efficiency ferredoxin (Fdx4509) selected as a comparative control, as well as the structures of P450–Fdx and FdR–Fdx electron-transfer complexes.

Our previous studies have shown that P450-Fdx recognition is primarily governed by electrostatic complementarity, particularly between acidic residues surrounding the Fdx iron–sulfur cluster and basic residues on the proximal face of P450 (48). Structural analysis of these predicted complexes showed that all six Fdxs bind to the proximal surface of CYP142A12, consistent with canonical class I P450 electron–transfer complexes (48). Electrostatic surface analysis of the protein–protein interaction interfaces further revealed that the proximal face of CYP142A12 possesses a prominent positively charged region, whereas the P450-interacting surfaces of Fdxs with high P450-supporting activity, such as *Sel*Fdx1499 and Fdx4443, exhibit strongly negatively charged surfaces around their iron–sulfur cluster (Fig. S20). In contrast, Fdx2666 and Fdx3040 show moderate negative charge, while Fdx4515 and Fdx4509 exhibit weakly negative surfaces. Although all six Fdxs can interact with CYP142A12, differences in acidic residue distribution lead to distinct predicted binding orientations. Notably, this electrostatic feature correlates well with the experimentally measured P450-supporting activities (Figs. 4*B* and 5), a more negatively charged surface might provide greater opportunities for forming optimal interfaces with P450 enzymes, thereby enabling more efficient electron transfer.

Beyond electrostatics, electron-transfer efficiency is strongly affected by the distance between the redox centers. AlphaFold3-predicted models revealed that high-activity complexes *Sel*Fdx1499-CYP142A12, Fdx4443-CYP142A12, and Fdx2666-CYP142A12 exhibit iron–sulfur cluster to heme iron distances of 13.7, 14.0, and 14.5 Å, respectively. In contrast, lower-activity complexes Fdx3040-CYP142A12, Fdx4515-CYP142A12, and Fdx4509-CYP142A12 give the corresponding distances of 15.5 Å, 15.7 Å, and 17.2 Å, respectively (Fig. S22). A similar distance-dependent trend was observed for FdR–Fdx complexes. Analysis of the FdR–Fdx complexes revealed that the *Sel*FdR0978–*Sel*Fdx1499 complex exhibits a notably short FAD-iron-sulfur cluster distance of 6.5 Å, whereas when FdR4662 paired with its cognate Fdxs, including Fdx4443, Fdx2666, Fdx3040, Fdx4515, and Fdx4509, the shortest distances between FAD and iron-sulfur clusters were 17.1 Å, 20.8 Å, 22.4 Å, 20.1 Å, and 20.4 Å, respectively (Fig. S23).

To assess whether differences in redox partner efficiency correlate with variations in binding affinity, we quantitatively determined the dissociation constants (*K*_D_) of CYP142A12 with five Fdx proteins using spectral titration assays (Fig. S21 and Table 2). CYP142A12 exhibited the highest binding affinity for its endogenous partner Fdx4443, followed by Fdx2666 and *Sel*Fdx1499 (Table 2). Notably, although *Sel*Fdx1499 emerged as the most efficient exogenous partner due to favorable electrostatic features and a short electron transfer distance comparable to the native Fdxs, its binding affinity was nearly twofold weaker than that of the endogenous Fdx4443. This distinction highlights that the specific co-evolution of the native Fdx4443–CYP142A12 pair might confer a unique affinity advantage.

**Table 2:** Binding affinities of ferredoxins toward CYP142A12

Cytochrome P450	Ferredoxin	*K*_*D*_ (μM)
CYP142A12	Fdx4443	31 ± 10
	Fdx2666	47 ± 17
	*Sel*Fdx1499	58 ± 28
	Fdx3040	83 ± 33
	Fdx4515	104 ± 46

Taken together, the magnitude of the negatively charged surface on Fdx, the iron-sulfur cluster to heme-iron distance in P450–Fdx complexes, the FAD-cluster distance in FdR–Fdx complexes, and the P450-Fdx binding affinity appear to be the key determinants that co-mediate the electron transfer efficiency in P450 catalytic systems.

## Discussion

Sterol side-chain degradation in *Mycobacterium* species proceeds through a class I P450-mediated C26 hydroxylation step that initiates *β*-oxidative chain shortening. Although the overall pathway has been delineated, the identities of the cognate FdR/Fdx redox partners that support sterol-metabolizing P450s have remained largely unexplored. Owing to its remarkable sterol biotransformation capacity and genetic tractability, *M. neoaurum* serves as an excellent model system for addressing these fundamental questions. In this study, we identified five sterol-oxidizing P450 enzymes (CYP125A76, CYP125A77, CYP125A78, CYP142A12, and CYP124A1) from *M. neoaurum* ZC-1 that catalyze terminal oxidation of sterol side chains, a crucial step in cholesterol catabolism. Here, by reconstructing the redox network of *M. neoaurum* ZC-1 *in vitro*, we identify FdR4662/Fdx4443 as a preferred electron-transfer chain for five sterol-oxidizing P450s. Interestingly, genomic mapping shows that the genes for FdR4662 and Fdx4443 are not colocalized with the five sterol-oxidizing P450 genes (Fig. 1), yet this pair consistently supported the highest conversions across multiple enzymes and substrates. Such functional specificity in a dispersed redox repertoire suggests that *M. neoaurum* can flexibly rewire electron flow through protein-protein recognition, enabling rapid maintenance of efficient sterol catabolism.

Biochemical assays and sequence analysis showed that FdRs in *M. neoaurum* predominantly prefer NADH, with eight harboring the characteristic NADH-binding motif. In addition, we identified several atypical NADH-preferring FdRs, providing markers for annotating uncharacterized FdRs and guiding redox-partner engineering in the future. Mechanistically, sterol side-chain degradation is a catabolic process that elevates the intracellular NADH pool. Routing NADH into P450 catalysis *via* NADH-preferring FdRs would enhance oxidation throughput while conserving NADPH for biosynthesis and antioxidant defense. This “cofactor economy” reflects the established division of labor between NADH (catabolism, ATP generation) (69) and NADPH (anabolism, ROS detoxification) (70) and likely represents a key adaptive strategy in sterol-rich environments. Such optimization of redox balance may explain the exceptional sterol biotransformation capacity of *M. neoaurum*. In other prokaryotic organisms, *Corynebacterium glutamicum* ATCC 13032 encodes three FdRs (two are NADH-preferring and one is NADPH-preferring); *Streptomyces coelicolor* A3 (2) contains four FdRs (three are NADH-preferring, and one is NADPH-preferring); and *Synechococcus elongatus* PCC 7942 carries a single NADPH-preferring FdR (50). These observations indicate that some organisms possess FdRs with both NADH and NADPH cofactor preferences and that FdR cofactor specificity might have evolved to match distinct metabolic lifestyles and biosynthetic capacities, reflecting the elegant balance between anabolism and catabolism. In particular, the photoautotrophic *S. elongatus*, dominated by anabolic metabolism, employs a single NADPH-preferring FdR. Clarifying such preference is critical for understanding P450 redox networks and guiding cofactor-based engineering, which are essential for the rational optimization and engineering of industrial strains.

Owing to the intrinsically weak protein–protein interactions between P450 enzymes and their redox partners, together with the rapid inactivation of Fdx, acquiring cocrystallized structures of P450–Fdx and FdR–Fdx complexes remains highly challenging (48, 71, 72). Consequently, the limited number of available complex structures has long hindered detailed mechanistic understandings of electron transfer between redox partners and P450s. To date, only a few complex structures have been reported, including the Pdx^Asp38^–P450cam^Arg112^ complex (PDB code 2M56) (73) and *Bt*Adx-CYP11B2 fusion protein (PDB code 7M8I) (74). Notably, our predicted complex structures exhibit high structural similarity to these experimentally resolved models, supporting that electrostatic complementarity plays a key role in P450-Fdx recognition. A more negatively charged surface on Fdx is likely to increase the probability of forming an optimal interaction interface with P450 enzymes, thereby facilitating efficient electron transfer. Furthermore, the proper distances between Fdx and P450 (with high binding affinity), as well as between FdR and Fdx, are also the key factors determining the overall electron transfer rate and the catalytic efficiency of P450s. By integrating AlphaFold3-based structure prediction with structural analysis, this study provides new insights into the molecular determinants of redox partner selectivity and electron-transfer efficiency and establishes a framework for rational design of highly efficient P450 catalytic systems. In addition, a higher binding affinity between P450 and Fdx could further enhance catalytic efficiency.

Our findings also have practical implications. Overexpressing FdR4662/Fdx4443 could enhance P450-driven steroid transformations in industrial strains, while the structural “rules” identified here may guide the rational selection and engineering of redox partners for other P450s. In addition, directed evolution of moderate-activity P450s (*e.*g., CYP125A78 and CYP124A1) combined with optimized redox partners may yield next-generation biocatalysts. Similar strategies have already succeeded in *E. coli* and *M. neoaurum*, where redox partner optimization increased steroid yields several-fold (75, 76, 77). Further improvements are expected by boosting NADH supply and recycling and by alleviating ROS pressure, thereby enhancing the whole-cell production of AD, androsta-1,4-diene-3,17-dione, and 9-hydroxyandrost-4-ene-3,17-dione.

## Experimental procedures

### Materials

Sterol compounds were purchased from Aladdin and Sigma Aldrich. Antibiotics were obtained from Solarbio. High-fidelity enzymes PrimeSTAR Max DNA Polymerase R045Q and 2 × MultiF Seamless Assembly Mix were acquired from TaKaRa Bio Inc. and Abclonal, respectively. The bacterial genomic DNA extraction kit was purchased from Tiangen. Kits for gel extraction and plasmid mini-preparation were obtained from Omega Bio-Tek. The FlexiRun pre-made gel solution for SDS-PAGE analysis was provided by Beyotime Biotech Inc. The *E. coli* BL21(DE3) strain and plasmid vectors for expressing *Sel*Fdx1499 and *Sel*FdR0978 proteins were retained in this laboratory.

### Construction of expression vectors

Genomic DNA from *M*. *neoaurum* ZC-1 strain was extracted to serve as a cloning template. The 32 P450 genes were initially subcloned into the pET22b vector for expressing the *N*-His_6_-tagged recombinant proteins. The genes encoding insoluble proteins, including 29 P450 enzymes, 14 Fdxs, and 11 FdRs, were codon-optimized according to the codon preference of *E. coli*. Specifically, the coding genes of CYP142A12, CYP124A1, CYP124P3, CYP125A76, and CYP125A77 were individually subcloned into the pET32a vector, leading to *C*-His_6_-tagged proteins. The remaining P450s, Fdx, and FdR genes were subcloned into the pMAL-c5E vector for expressing the fusion proteins with an *N*-terminal maltose-binding protein tag and a *C*-terminal hexahistidine tag. The accuracy of all constructs was confirmed through DNA sequencing by Sangon Biotech.

### Protein expression and purification

All P450s and redox partners were expressed in *E. coli* BL21(DE3) and purified *via* Ni-NTA affinity chromatography. Briefly, a single colony of certain recombinant strain was inoculated into LB broth and grown at 37 °C with shaking at 220 rpm overnight. The culture was then transferred into 0.5 L Terrific Broth medium at an inoculation ratio of 1:100 (*v/v*) in a 2 L conical flask and incubated at 37 °C until the A_600_ reached 0.6 to 0.8. Protein expression was induced with 0.4 mM IPTG. After induction, the temperature was reduced to 16 °C, and the cells were cultured for an additional 20 h before being centrifuged at 6000*g* for 10 min to pellet the cells (41).

Both seed culture medium and fermentation culture medium required the addition of the antibiotic ampicillin (100 mg/ml). For P450 expression, the fermentation culture medium was supplemented with a rare salt solution (25 μM FeCl_3_⋅6H_2_O, 4 μM ZnCl_2_, 2 μM CoCl_2_⋅6H_2_O, 2 μM Na_2_MoO_4_⋅2H_2_O, 2 μM CaCl_2_, 3 μM CuSO_4_, and 2 μM H_3_BO_3_) (78, 79). In addition, 0.5 mM *δ*-aminolevulinic acid was added as a heme precursor.

The following protein purification was performed using Ni-NTA affinity chromatography as previously described (80, 81). The final purified proteins were flash-frozen in liquid nitrogen and stored at −80 °C for later use. P450 concentrations were determined from CO•Fe(II) *versus* Fe(II) difference spectra (ε_450-490nm_ = 91,000 M^−1^ cm^−1^) (68). The concentrations of Fdxs and FdRs were determined spectrophotometrically after appropriate dilution. The absorbance at 280 nm (A_280_) of 1 μl of each diluted sample was measured using a NanoDrop 2000 spectrophotometer (Thermo Fisher Scientific), and the protein concentrations were then calculated based on the dilution factor and the extinction coefficient.

### Ferredoxin reductase activity assay

To evaluate the activity of FdRs, we employed the redox indicator sodium DCIP as an electron acceptor. During the reduction reaction, blue DCIP accepts electrons and is reduced to a colorless form, enabling quantitative analysis by monitoring the decrease in absorbance at 600 nm using a SpectraMax^M2^ spectrophotometer (Molecular Devices).

Thus, the reduction activity of FdR was indirectly measured by monitoring the rate of DCIP reduction. The reaction was conducted in 50 mM potassium phosphate buffer (pH 7.4), with a reaction system comprising 100 nM FdR and 100 mM DCIP. The reaction was initiated by adding 500 μM NAD(P)H and measured within 1 min. The concentrations of DCIP at different time points were calculated using the extinction coefficient (ε_600_ = 21,800 M^−1^ cm^−1^) (82). Electron donor preference was also investigated by using NADH to replace NADPH for reaction initiation.

#### ***Cytc*reduction assays**

The electron transfer efficiency of different combinations of Fdx and FdR (12 Fdxs × 10 FdRs) was measured as previously described (48). This was done by monitoring the increase of reduced cyt *c* at 550 nm using the extinction coefficient of 21,000 M^-1^ cm^-1^ with a UV-visible spectrophotometer (Varian) (83, 84). The reaction mixture contained 50 μM cytochrome *c*, 5 μM FdR, and 10 μM Fdx in 50 mM potassium phosphate buffer (pH 7.4) (85). The reactions were initiated by adding 500 μM NAD(P)H. Steady-state kinetic analyses were performed using OriginPro 8.5 program.

### P450 enzymatic assays

To evaluate the activity of the five P450 enzymes in combination with 12 Fdxs and 10 FdRs, standard reactions were conducted in 50 mM potassium phosphate buffer (pH 7.4). Each reaction contained 2 μM P450, 20 μM Fdx, 10 μM FdR, 200 μM substrate, 2.5 mM NAD(P)^+^, and an NAD(P)H regeneration system (10 mM glucose and 2 U glucose-6-phosphate dehydrogenase) in a final reaction volume of 100 μl. The P450:Fdx:FdR ratio of 1:10:5 was selected to optimize substrate conversion efficiency (48). All reactions were carried out at 30 °C for 12 h. Negative control experiments were performed using boiling-inactivated enzymes. The enzymatic reactions were terminated by adding 1 M HCl in one-10th of the reaction volume. For reactions with 7-dehydrocholesterol and 4-cholesten-3-one as substrates, equal volumes of methanol were added; while for the reactions using cholesterol as substrate, 150 μl of ethyl acetate was added. The mixtures were vortexed for 10 min and centrifuged for 10 min to separate the extracts. For cholesterol product detection, the extract was derivatized with an equal volume of *N*,*O*-bis (trimethylsilyl) trifluoroacetamide with 1% trimethylchlorosilane at 72 °C for 30 min. Analytes were identified and quantified using HPLC, GC, liquid chromatography-mass spectrometry, or gas chromatography-mass spectrometry. All reactions were performed in triplicate, with statistical analyses performed using Student’s *t* test or analysis of variance (86).

### P450 substrate-binding assay

The purified P450 enzyme was diluted to a final concentration of 1 μM and titrated with 1 μl aliquots of substrate solution at concentrations ranging from 0.1 to 1 mM, pre-dissolved in hydroxypropyl-*β*-cyclodextrin. A control titration was conducted using 45% hydroxypropyl-*β*-cyclodextrin without substrate (56). Difference spectra were recorded at room temperature on a Molecular Devices SpectraMax^M2^ spectrophotometer, scanning within the wavelength range of 350 to 500 nm. The difference absorbance (ΔA), defined as *A*_peak 390 nm_ − *A*_trough 420 nm_, was calculated from at least duplicated measurements and plotted against substrate concentrations. Data were fitted to the hyperbolic equation ΔA = A_max_ × [S]/(*K*_*D*_ + [S]) using OriginPro 9.0, where A_max_ represents the maximum absorbance shift at saturation, [S] denotes the substrate concentration, and *K*_*D*_ is the apparent dissociation constant of the enzyme-substrate complex (68).

### Spectral ferredoxin-binding assay

Spectral ferredoxin titrations were performed at room temperature in a desalting buffer containing 1 μM CYP142A12 and 1 μM cholesterol. The P450 solution was titrated with increasing concentrations of ferredoxin. Binding of ferredoxin to CYP142A12 induced spectral changes, and the absorbance difference was calculated as Δ*A* (*A*_peak 390 nm_ − *A*_trough 420 nm_). All measurements were performed in triplicate, and the dissociation constant (*K*_*D*_) was obtained by fitting the data to a hyperbolic equation Δ*A* = *A*max × [Fdx]/(*K*_*D*_ + [Fdx]) using OriginPro 9.0.

### Analytical methods

GC and gas chromatography-mass spectrometry analyses were conducted on an Agilent 7890B gas chromatographer and 1200 series instruments (Agilent Technologies Inc) equipped with an HP-5 capillary column (30 m × 0.25 mm, 2.5 μm). The program used for GC analysis was as follows: 90 °C for 3 min, the temperature was increased from 90 °C to 260 °C at 15 °C min^-1^, then to 275 °C at 1 °C/min for 13 min. HPLC and HPLC-HRMS analyses were carried out using a Thermo Ultimate 3000 (Thermo Fisher Scientific Inc.) and a Bruker impact HD high-resolution Q-TOF mass spectrometer (Bruker Corporation), respectively. Both analyses employed a Triart C18 column (5 μm, 2.1 mm × 100 mm, Thermo Fisher Scientific Inc), with mobile phase and detection wavelength optimized for specific target compounds. For 7-dehydrocholesterol and its products, detection was performed at 280 nm using a mobile phase consisting of solvent A (water + 0.1% formic acid) and solvent C (methanol). The gradient elution program was as follows: 0 to 1 min, 90% solvent C; 2 to 5 min, 90 to 100% solvent C; and 5 to 25 min, 100% solvent C, with a flow rate of 1.0 ml/min. For 4-cholesten-3-one, detection was performed at 240 nm using a mobile phase of solvent A and solvent B (acetonitrile) as the mobile phase. The gradient program was as follows: 0 to 1 min, 90% solvent B; 2 to 5 min, 90 to 100% solvent B; 5 to 25 min, 100% solvent B, with a flow rate of 1.0 ml/min.

## Data availability

All data supporting the findings of this study are available in the article and its supplementary information files.

## Supporting information

This article contains supporting information.

## Conflict of interest

The authors declare that they have no conflicts of interest with the contents of this article.
