# Supplementary material for: Supporting activities of cognate redox partners for sterol-metabolizing P450 enzymes in Mycobacterium neoaurum
Source: J Biol Chem. 2026 May 7;302(6):113116. doi: 10.1016/j.jbc.2026.113116 (PMC13254585; doi:10.1016/j.jbc.2026.113116)
Supplement: Supporting information [file mmc1.pdf]

Supplementary Information

Supporting activities of cognate redox partners for sterol-metabolizing P450 enzymes in *Mycobacterium neoaurum*

Yunjie Liu[1], Yue Zhao[1], Weihan Sun[1], Nian Li[2], Yunjun Pan[1], Li Ma[1,*], Shengying Li[1,3*]

[1] State Key Laboratory of Microbial Technology, Shandong University, Qingdao, Shandong 266237, China

[2] Melton International School, Qingdao, Shandong 266071, China

[3] Laboratory for Marine Biology and Biotechnology, Qingdao Marine Science and Technology Center, Qingdao, Shandong 266237, China

[*]Corresponding author: Li Ma (maliqd@sdu.edu.cn), Shengying Li (lishengying@sdu.edu.cn)

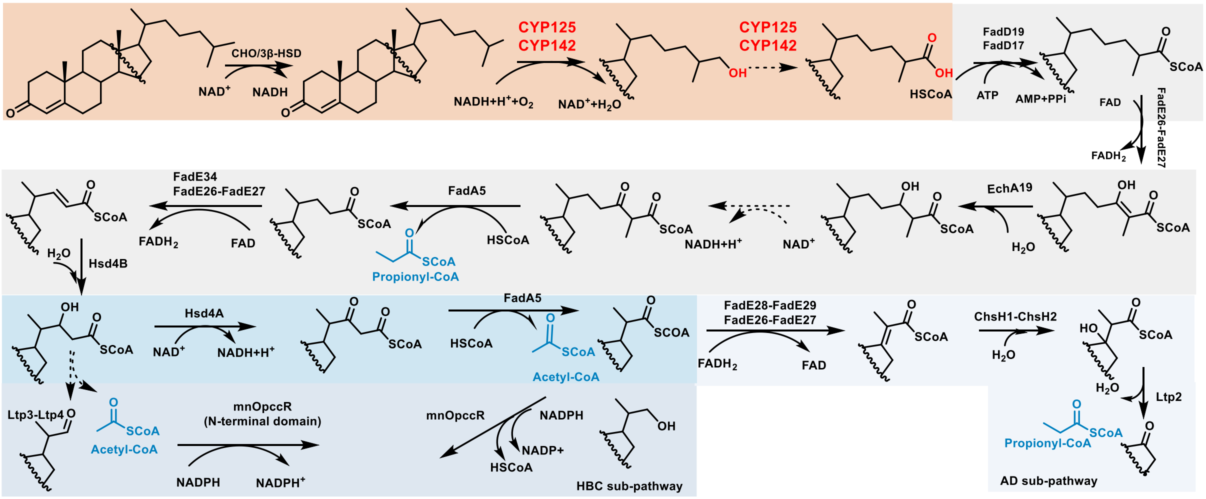

Figure S1. Cholesterol side-chain degradation pathways in *Mycobacteria*. The degradation pathway starts with terminal hydroxylation, followed by oxidation to a carboxyl group (highlighted in red). The resulting carboxylate is subsequently activated via CoA thioesterification. The pathway is further divided into the androstane derivative (AD) sub-pathway and the 2-hydroxy-hexa-2,4-dienoic acid (HBC) sub-pathway.

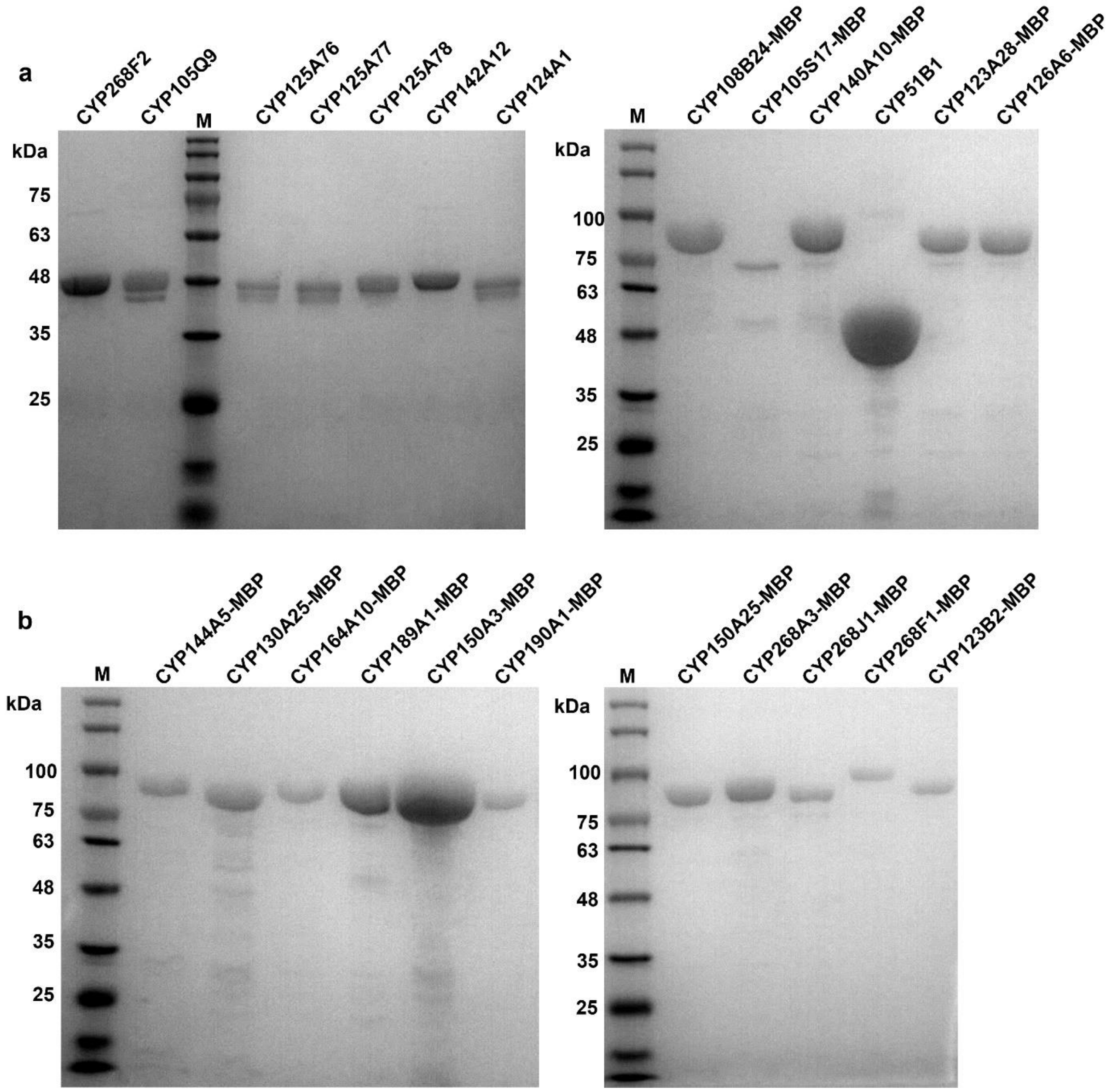

Figure S2. SDS-PAGE analysis of twenty-four purified P450 enzymes. The molecular weights of the eight P450 enzymes (CYP51B1, CYP268F2, CYP105Q9, CYP125A76, CYP125A77, CYP125A78, CYP142A1, and CYP124A1) range from 45-50 kDa, while the remaining P450 proteins fused with an *N*-terminal MBP tag have the molecular weights of 80-90 kDa.

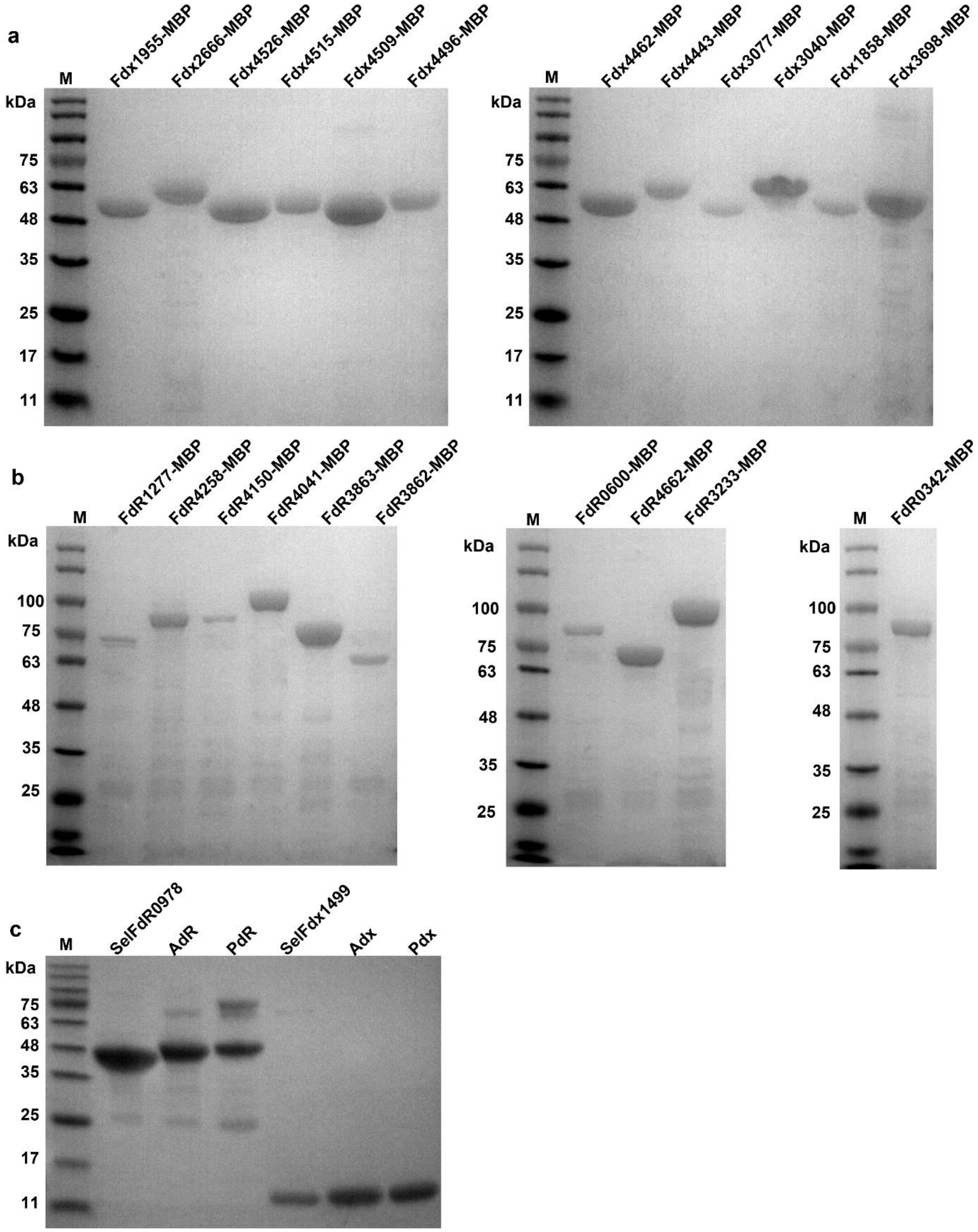

Figure S3. SDS-PAGE analysis of purified Fdxs and FdRs including 12 native Fdxs (a), 11 native FdRs (b), and 3 pairs of surrogate redox partners (c). Fdxs and FdRs were fused to an MBP tag on their *N*-termini.

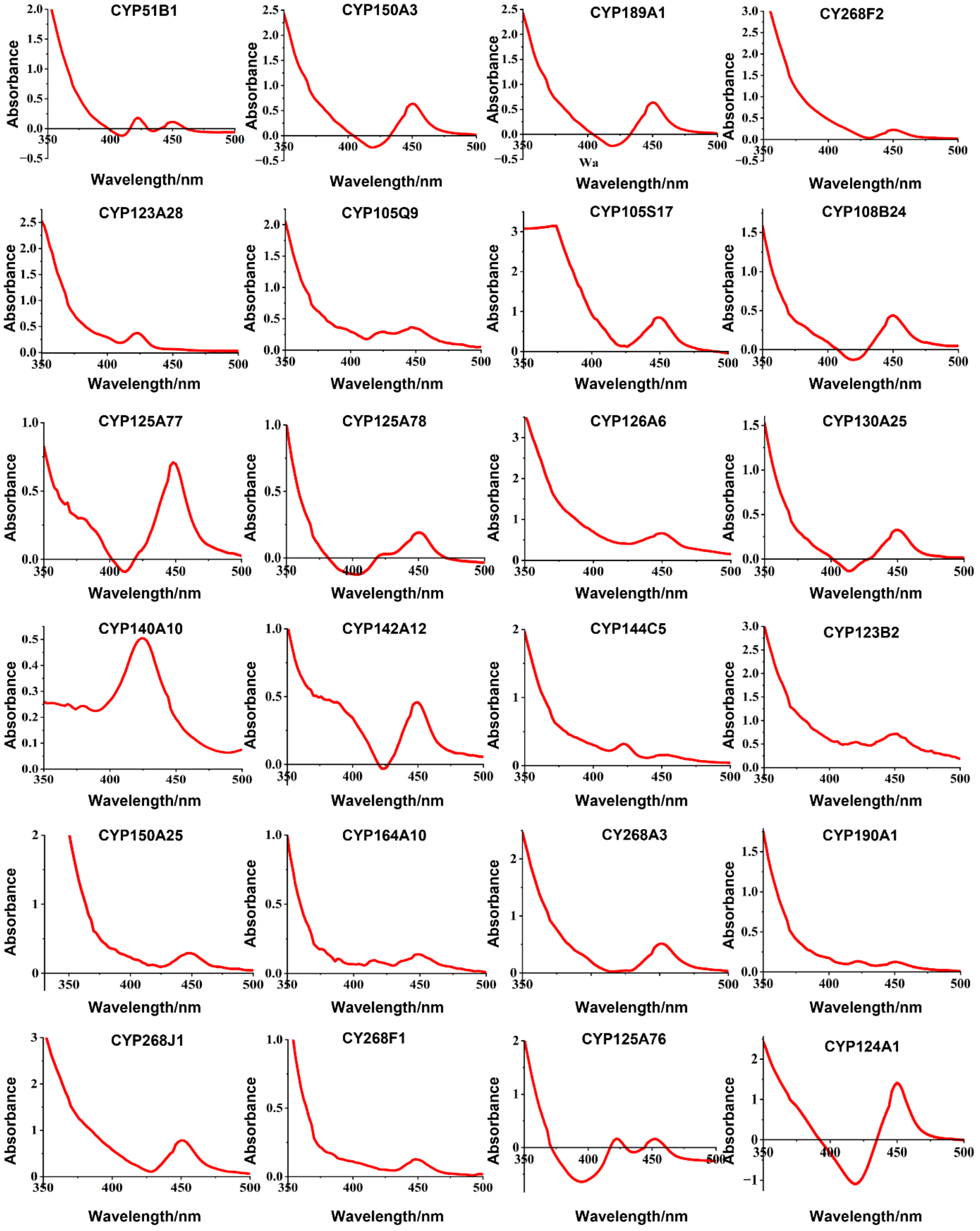

Figure S4. Characteristic absorption spectra of twenty-four purified P450 enzymes. *Solid line*, P450 absorbance spectra at the ferrous CO-bound state. These spectra were also employed to determine the concentrations of functional P450 enzymes using the extinction coefficient of $\varepsilon_{450\text{-}490} = 91{,}000\ M^{-1}cm^{-1}$(1, 2). The high absorbance observed in the UV region for some enzymes is likely due to the use of an excess of sodium dithionite during reduction.

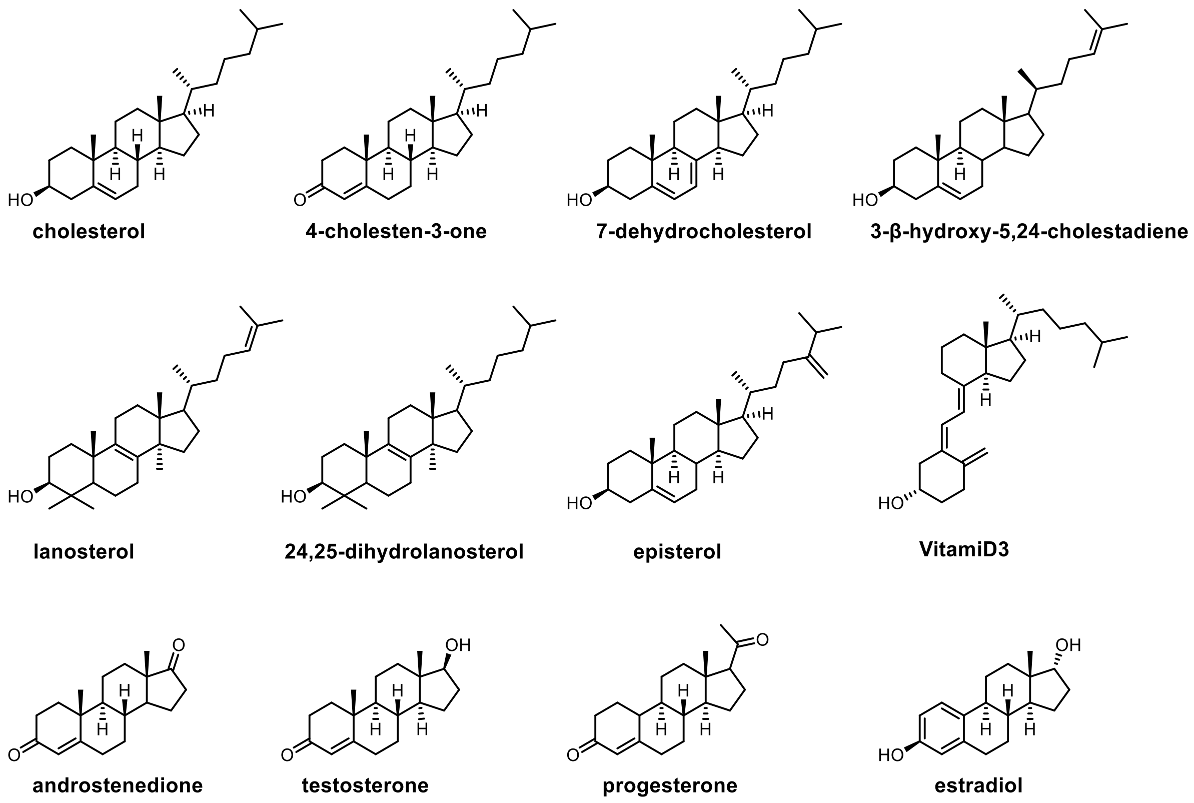

Figure S5. Sterol substrates used in this study.

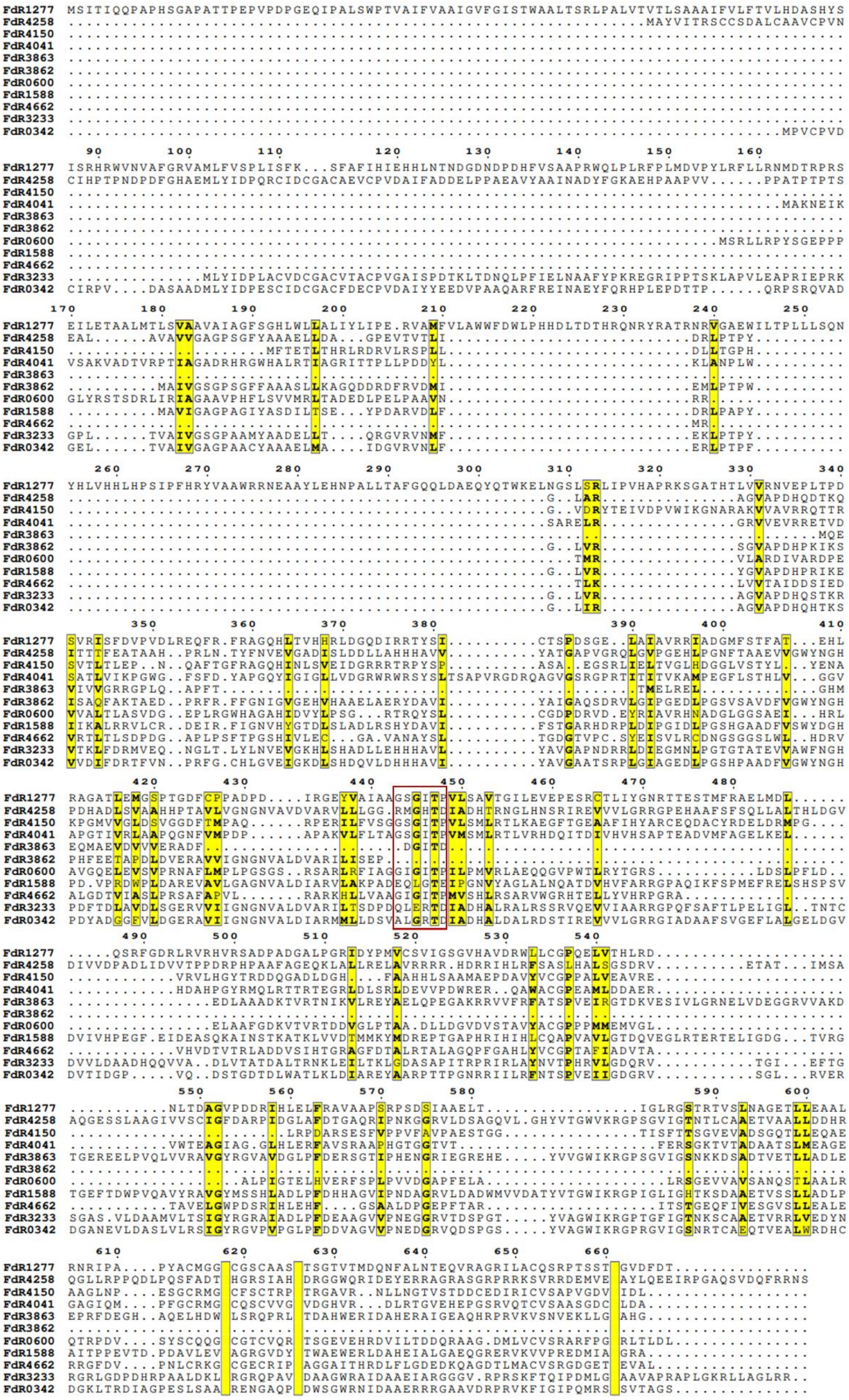

Figure S6. Protein sequence alignment of 11 FdRs. Sequence alignment was performed using the T-COFFEE online service, with the output generated by ESPript 3.0. A highly conserved "GXTX" motif is highlighted with a red box.

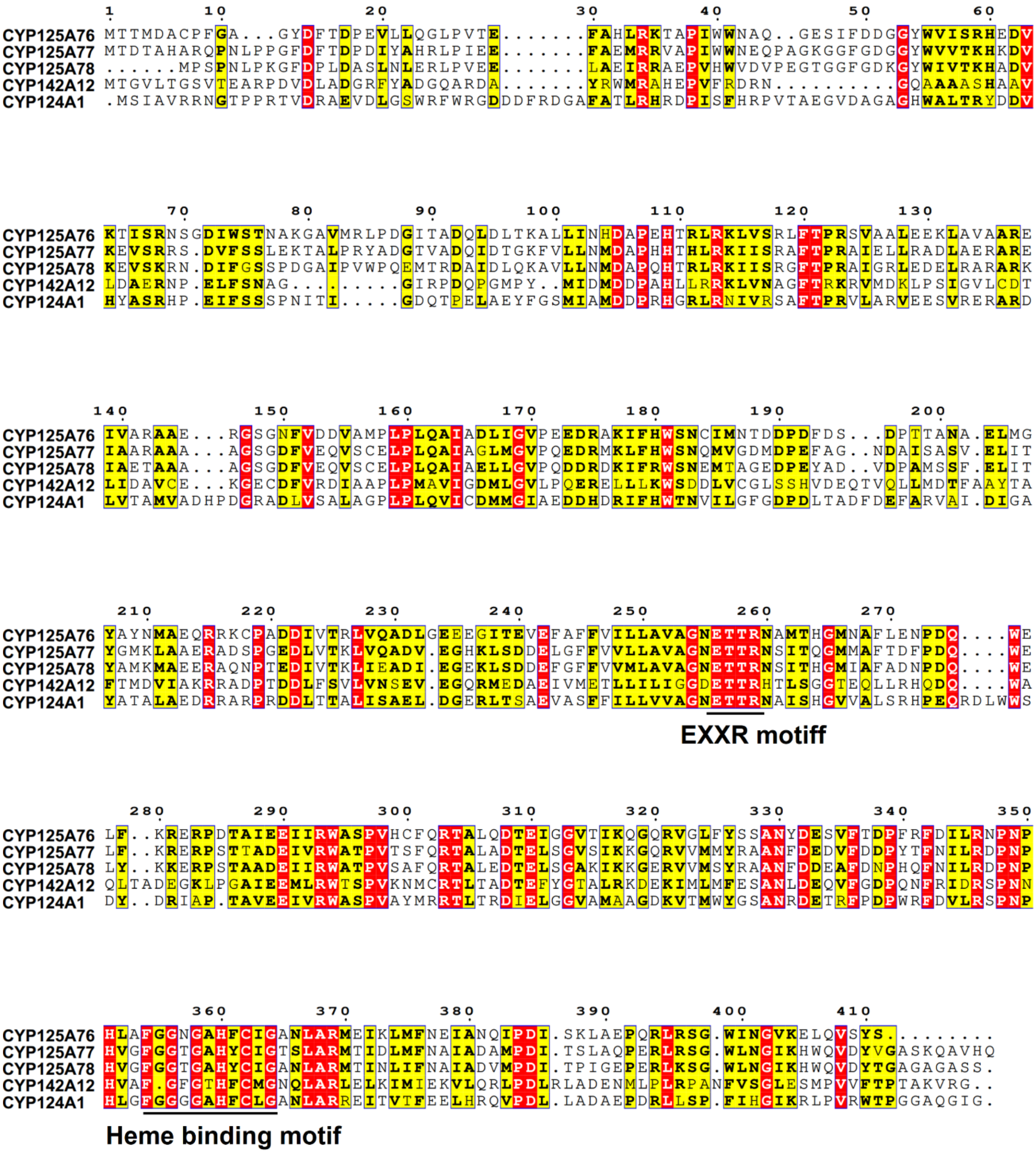

Figure S7. Protein sequence alignment of five P450 enzymes. Sequence alignment was performed using the T-COFFEE online service(3), with the output generated by ESPript 3.0(4). A highly conserved "FXXGXXXCXG" motif(5), characteristic of P450 enzyme is highlighted. The "EXXR" region, which is critical for proper protein folding and heme binding, is also highlighted.

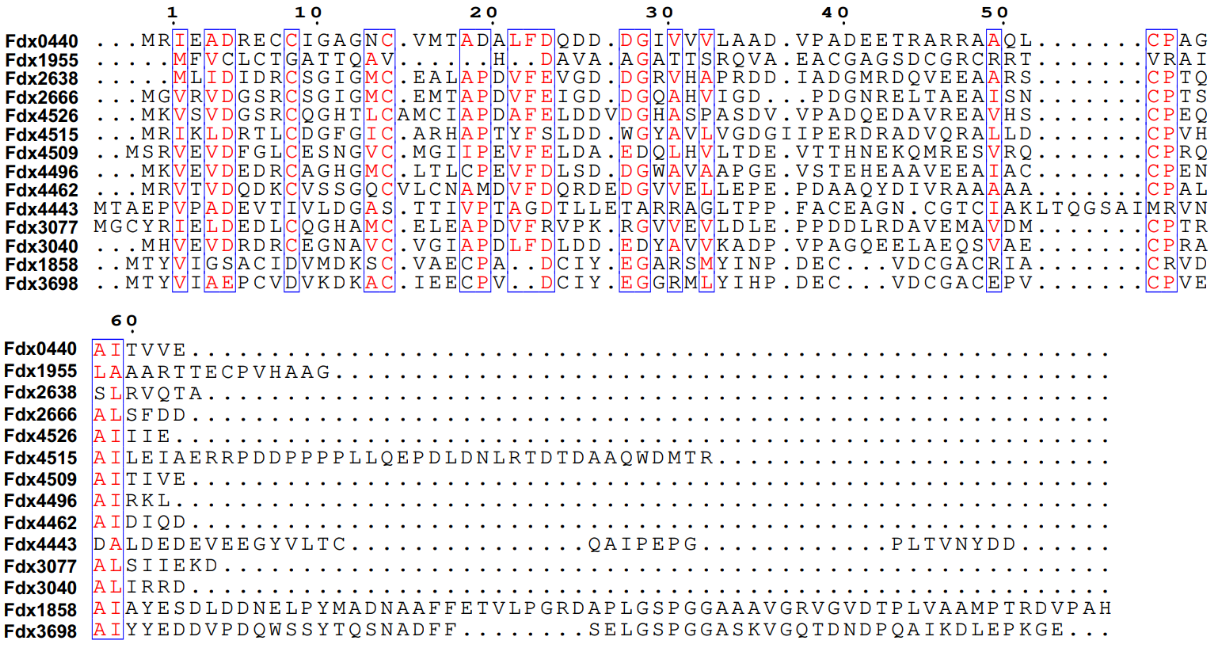

Figure S8. Protein sequence alignment of 12 Fdxs. Sequence alignment was performed using the T-COFFEE online service, with the output generated by ESPript 3.0.

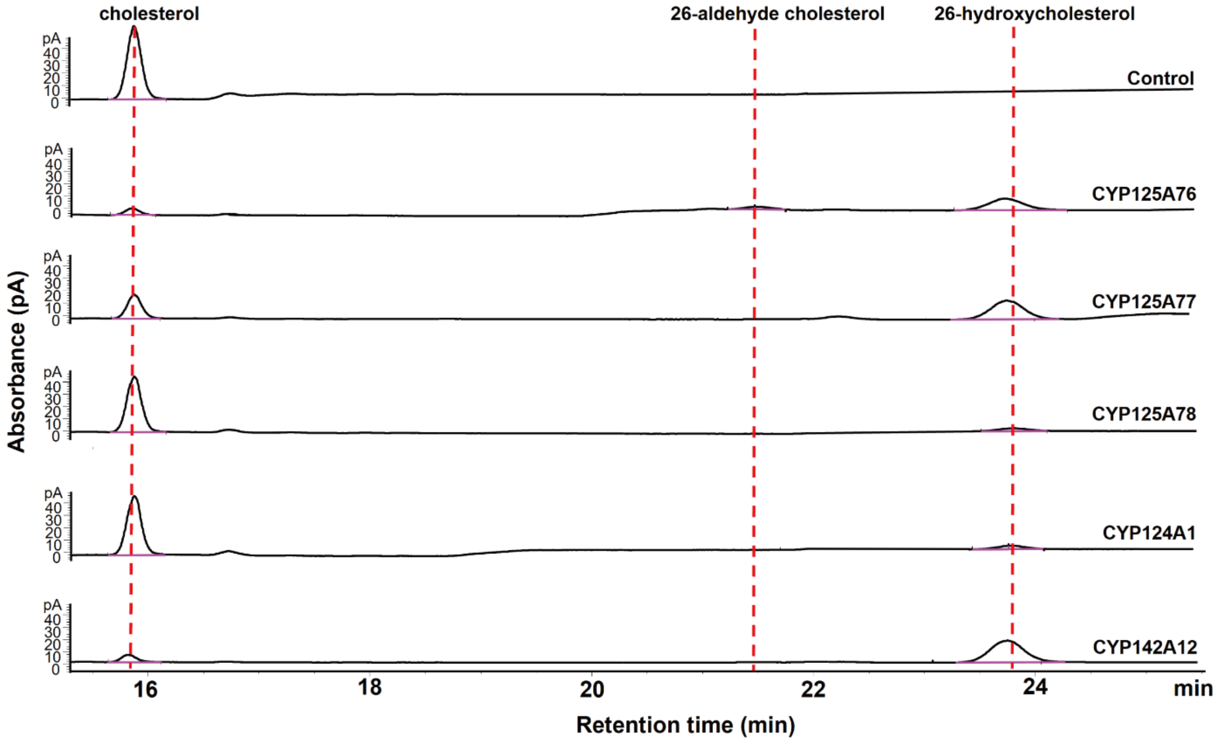

Figure S9. GC analysis of cholesterol oxidation reactions catalyzed by five P450 enzymes with FdR4662/Fdx4443 as redox partners.

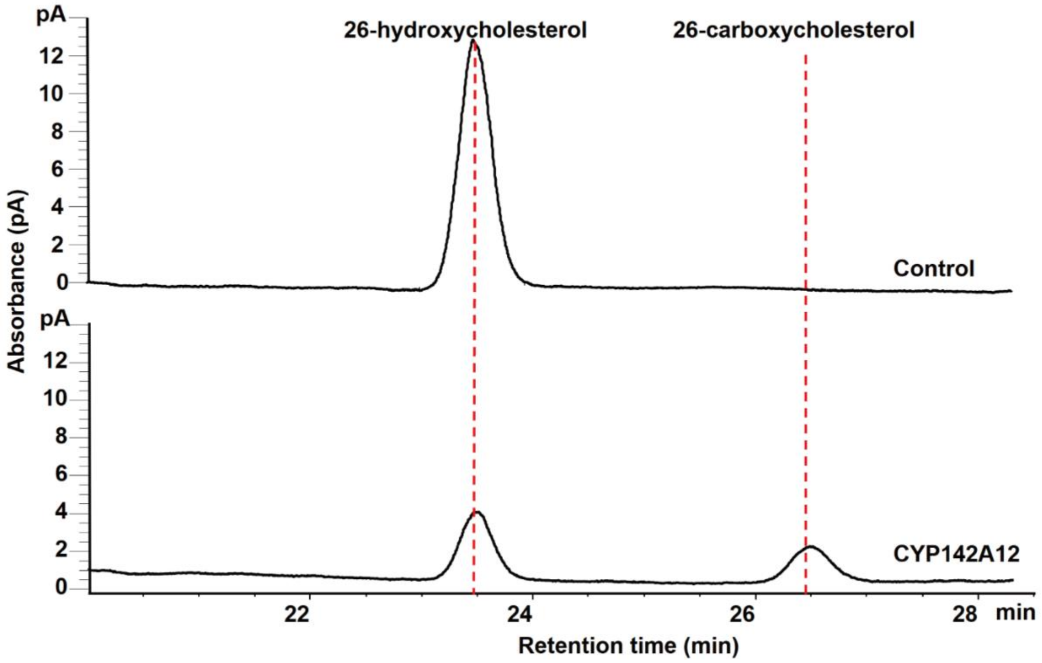

Figure S10. GC analysis of the 26-hydroxycholesterol oxidation reaction catalyzed by CYP142A12 with FdR4662/Fdx4443 as redox partners.

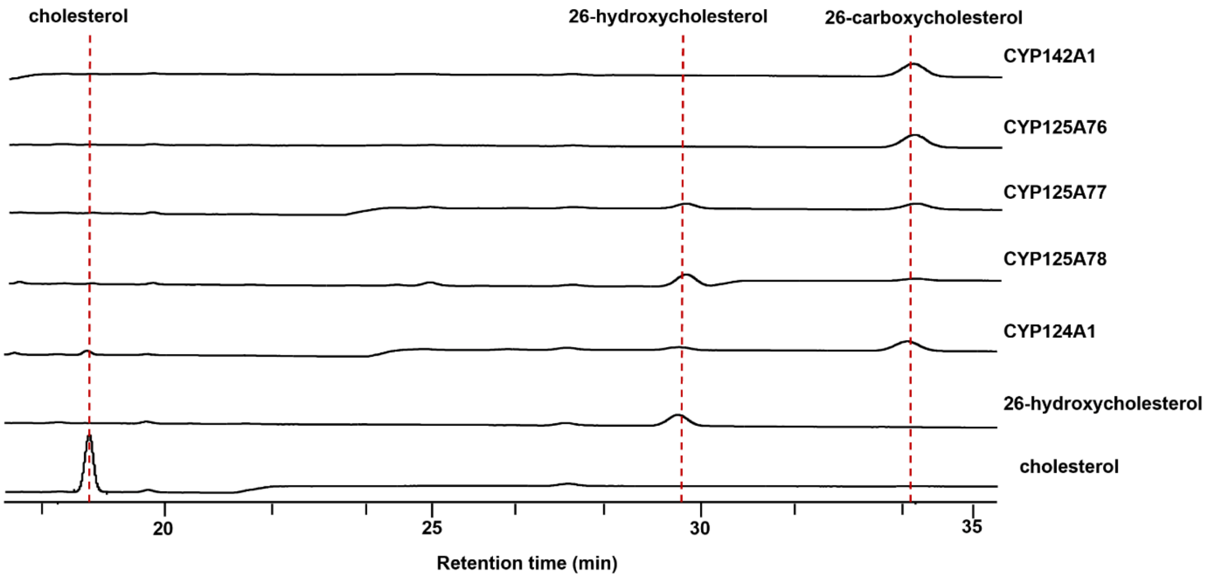

Figure S11. GC analysis of cholesterol oxidation reactions catalyzed by five P450 enzymes with *sel*FdR0978/*sel*Fdx1499 as redox partners.

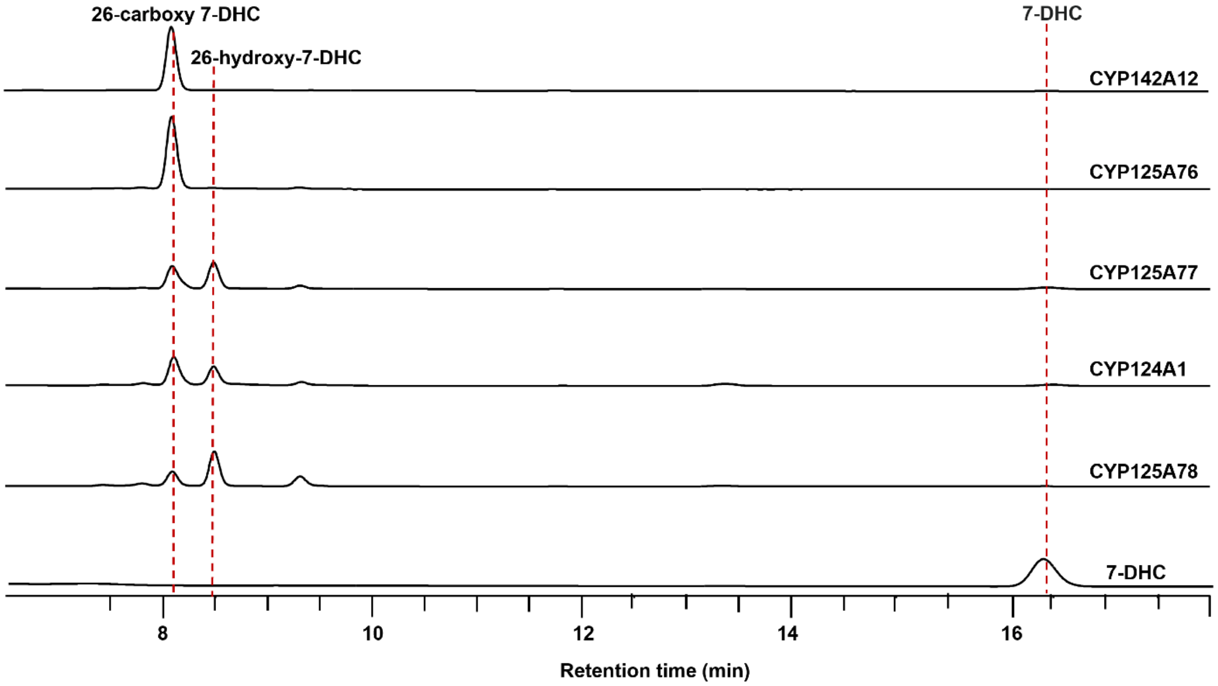

Figure S12. HPLC analysis of 7-dehydrocholesterol (7-DHC) oxidation reactions catalyzed by five P450 enzymes with *sel*FdR0978/*sel*Fdx1499 as redox partners.

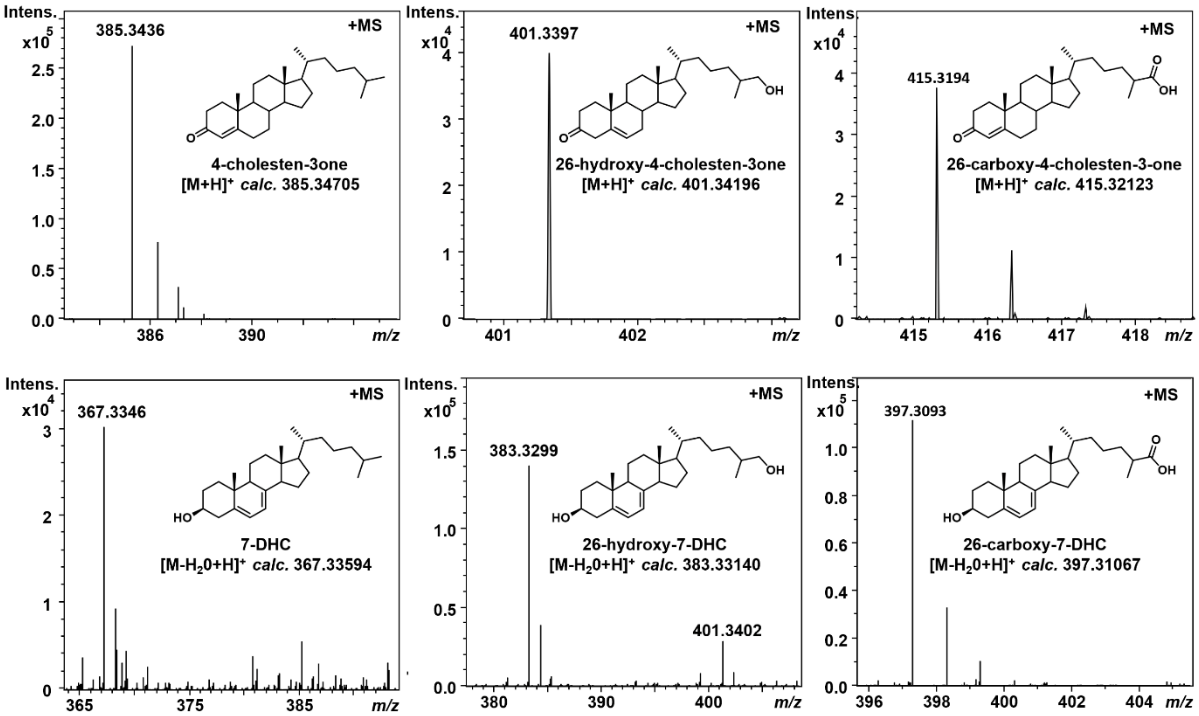

Figure S13. High-resolution mass spectra of substrates and products of P450 enzyme reactions.

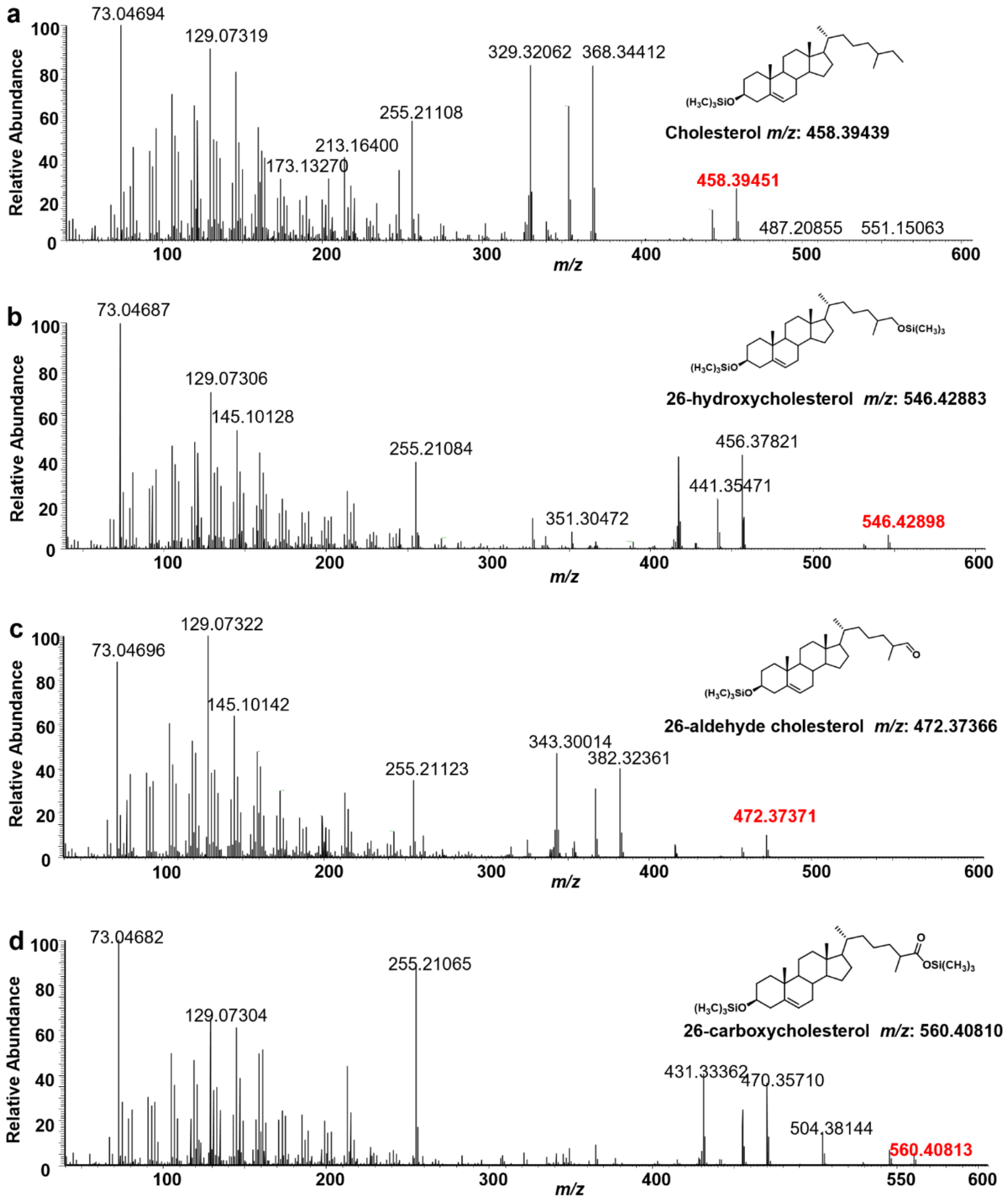

Figure S14. GC-MS analysis of BSTFA-derivatized cholesterol (a), 26-hydroxycholesterol (b), 26-aldehyde cholesterol (c), and 26-carboxycholesterol (d).

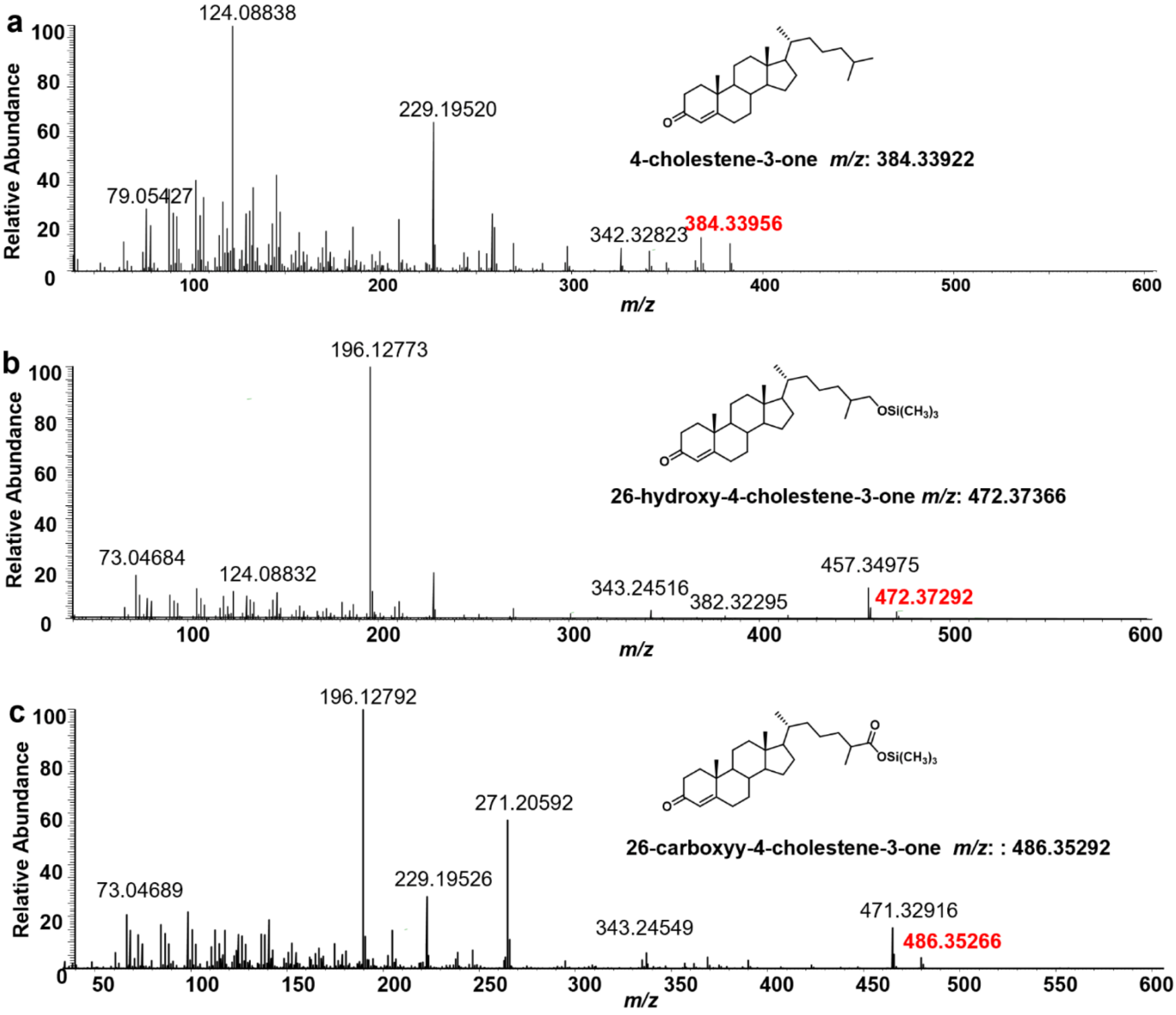

Figure S15. GC-MS analysis of BSTFA-derivatized 4-cholesten-3one (a), 26-hydroxy-4-cholesten-3one (b), and 26-carboxy-4-cholesten-3one (c).

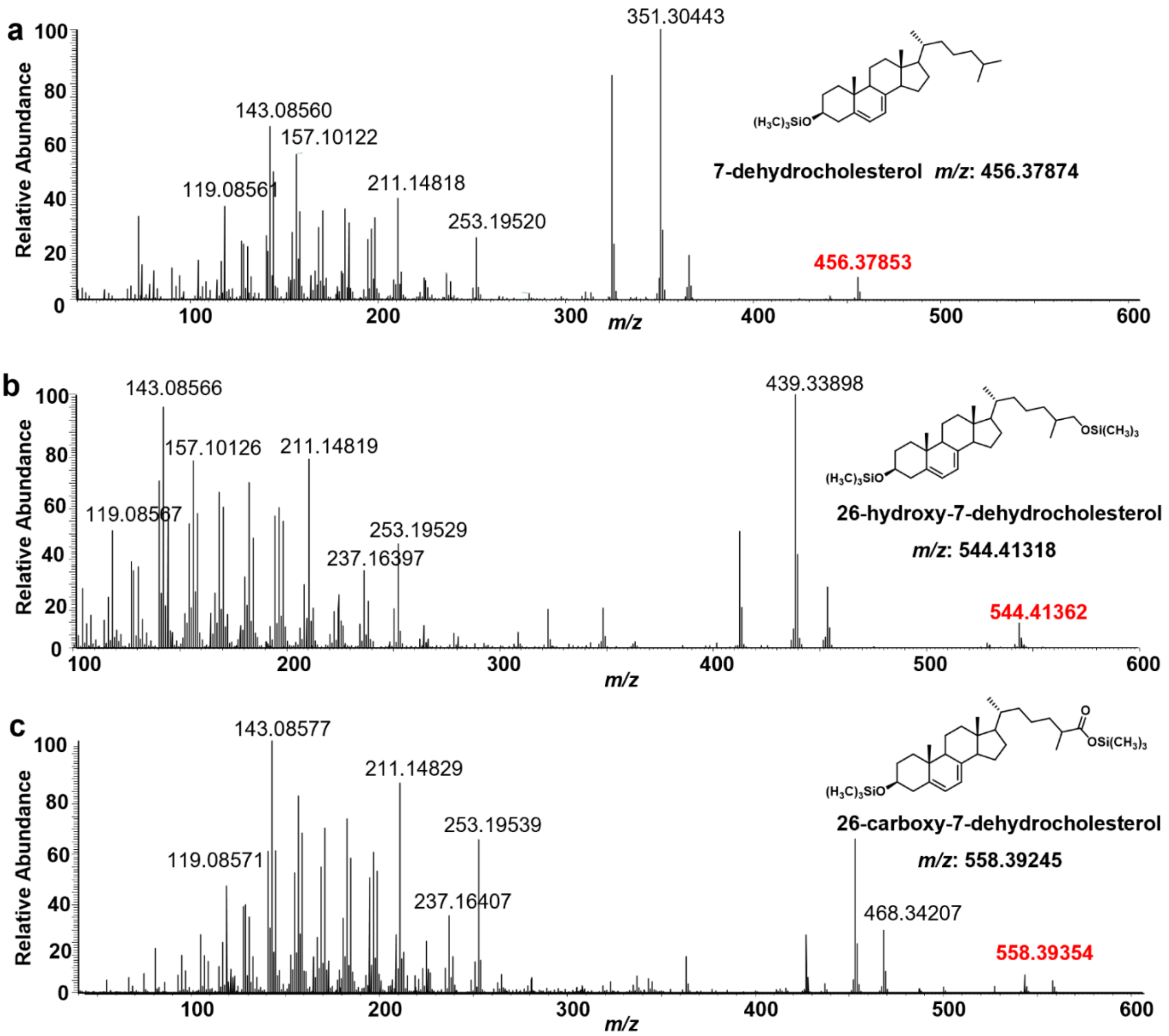

Figure S16. GC-MS analysis of BSTFA-derivatized 7-dehydrocholesterol (a), 26-hydroxy-7-dehydrocholesterol (b), and 26-carboxy-7-dehydrocholesterol (c).

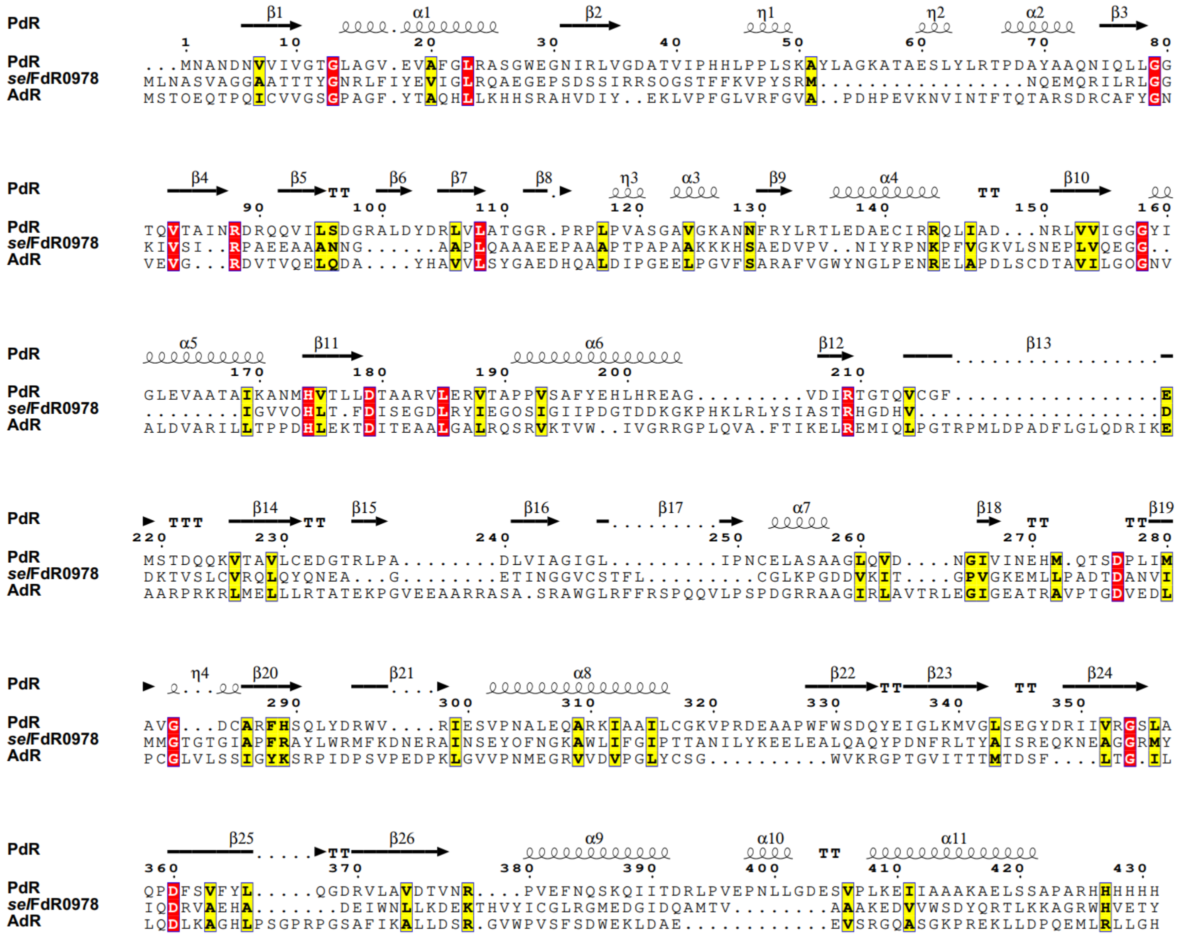

Figure S17. Protein sequence alignment of PdR, *sel*FdR0978 and AdR.

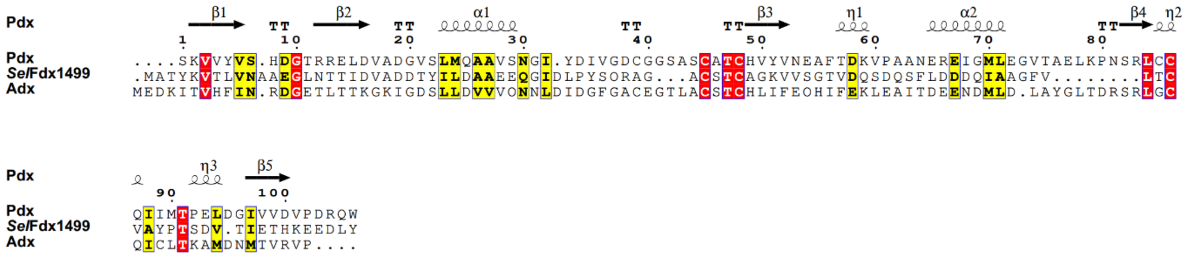

Figure S18. Protein sequence alignment of Pdx, *sel*Fdx1499, and Adx.

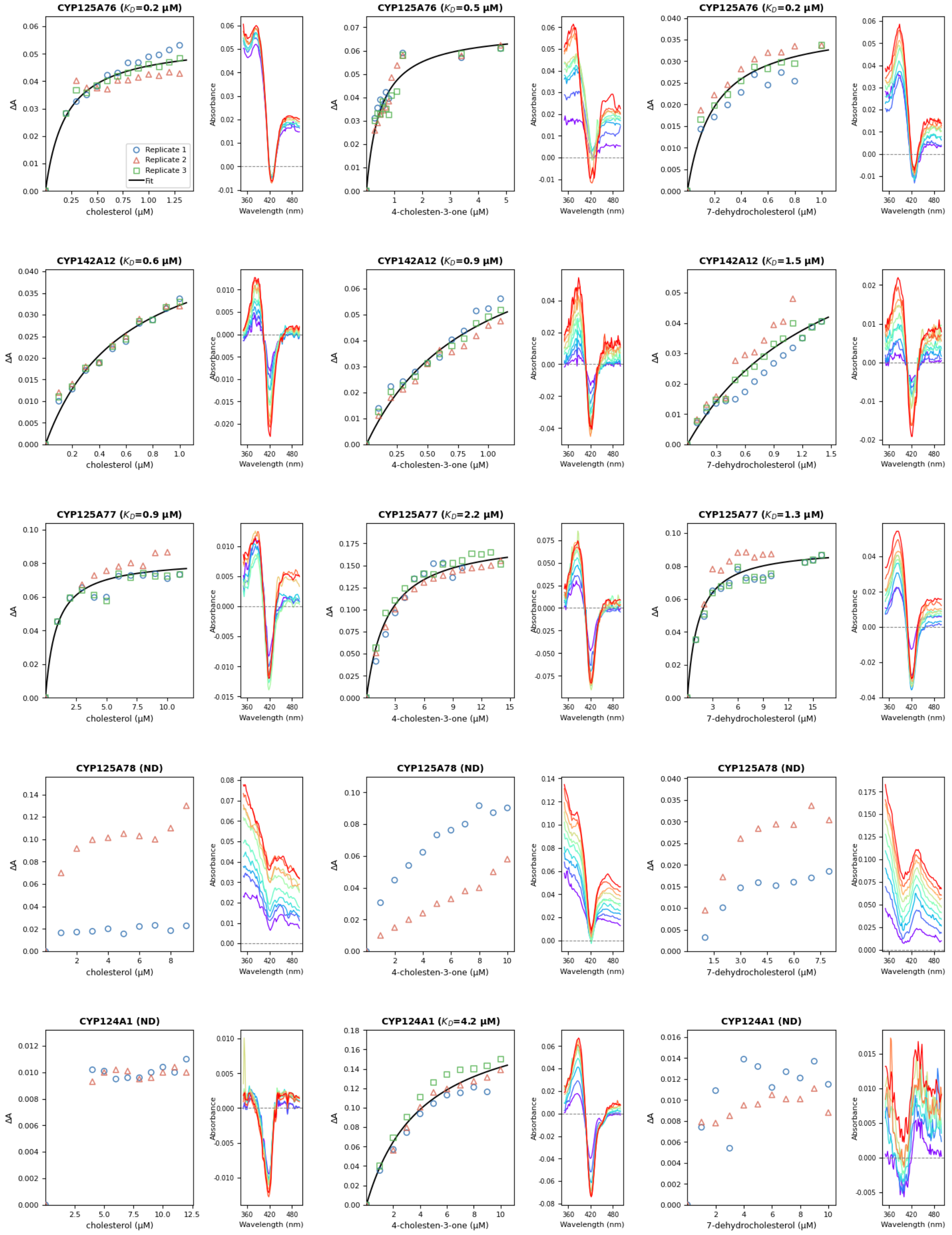

Figure S19. Substrate-binding curves of five P450 enzymes with cholesterol, 4-cholesten-3-one, and 7-dehydrocholesterol. Individual data points represent measurements in triplicate, and solid lines indicate the best-fit curves. The binding spectra are shown in the side panels. (ND: Not determined, as the binding curves could not be fitted).

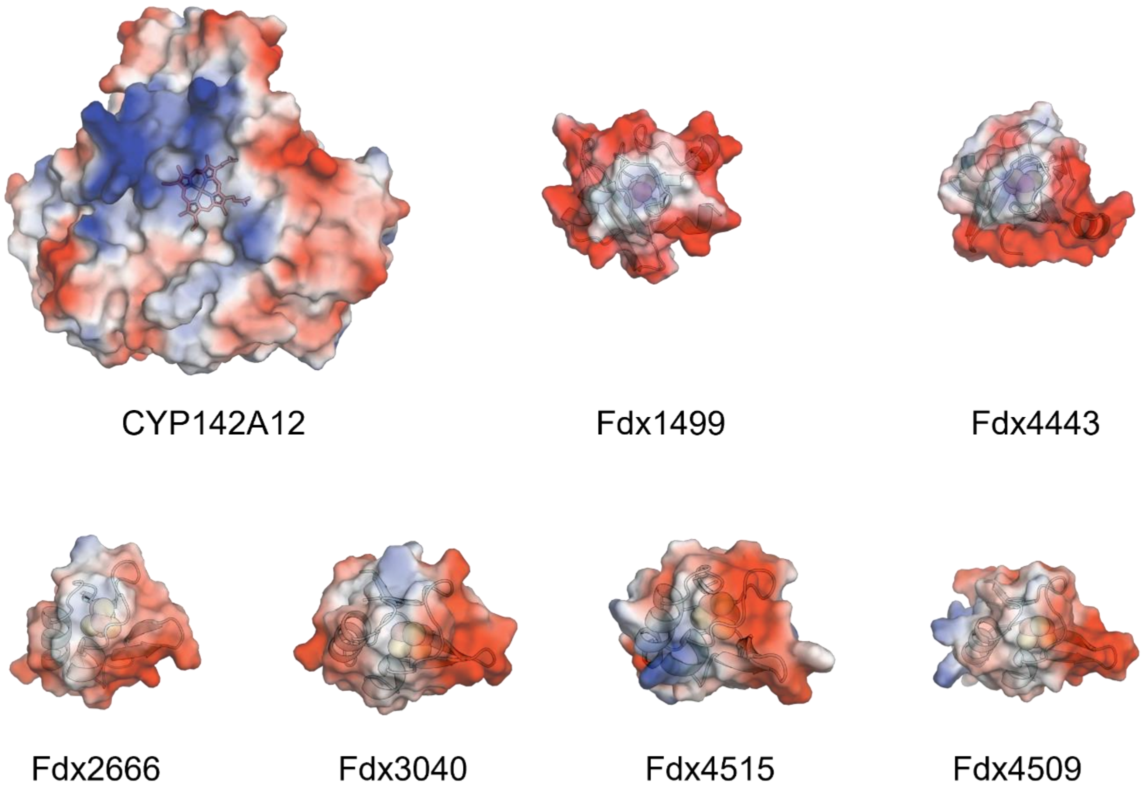

Figure S20. Electrostatic surface analysis of CYP142A12 and six Fdxs. The protein-protein interaction interfaces are shown for CYP142A12 (proximal face) and for each Fdx (the surface that interacts with the P450). Fdxs are shown in a same orientation to allow direct comparison. Positively and negatively charged regions are colored in blue and red, respectively.

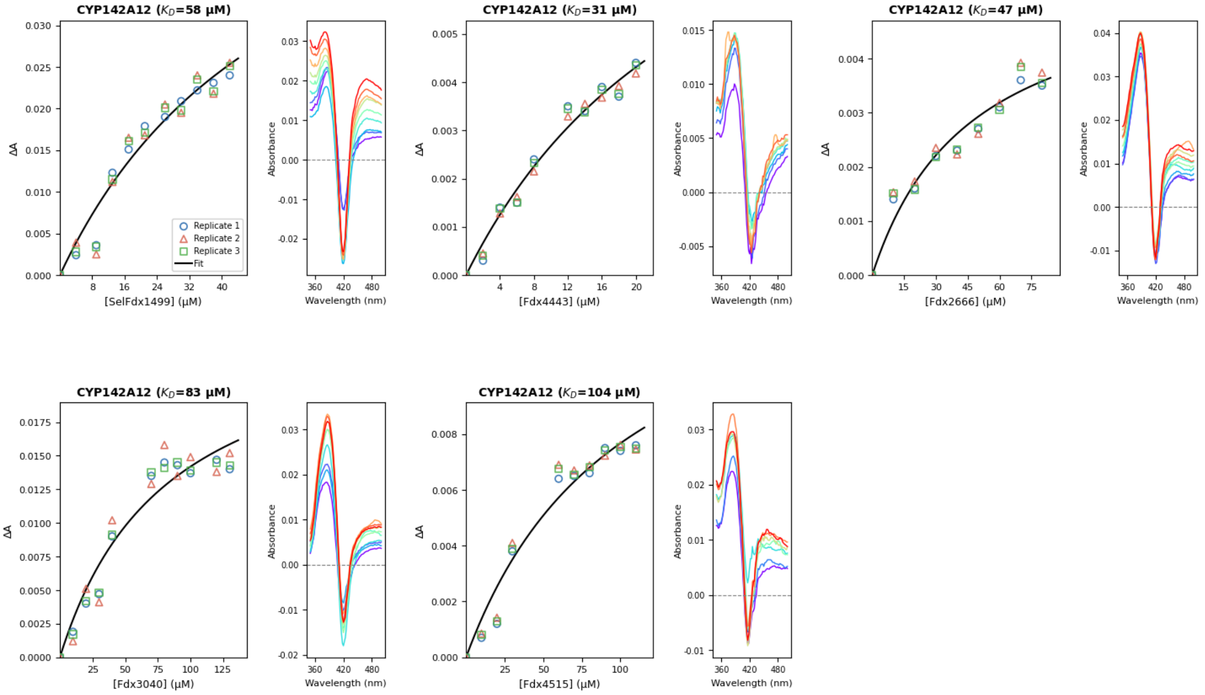

Figure S21. Protein-protein interaction binding curves of CYP142A12 with five ferredoxins (Fdx1499, Fdx4443, Fdx2666, Fdx3040, and Fdx4515). Individual data points represent measurements in triplicate, and solid lines indicate the best-fit curves. Corresponding spectral changes are shown in the side panels. The dissociation constants (K_D) were obtained by fitting spectral changes upon titration of ferredoxins.

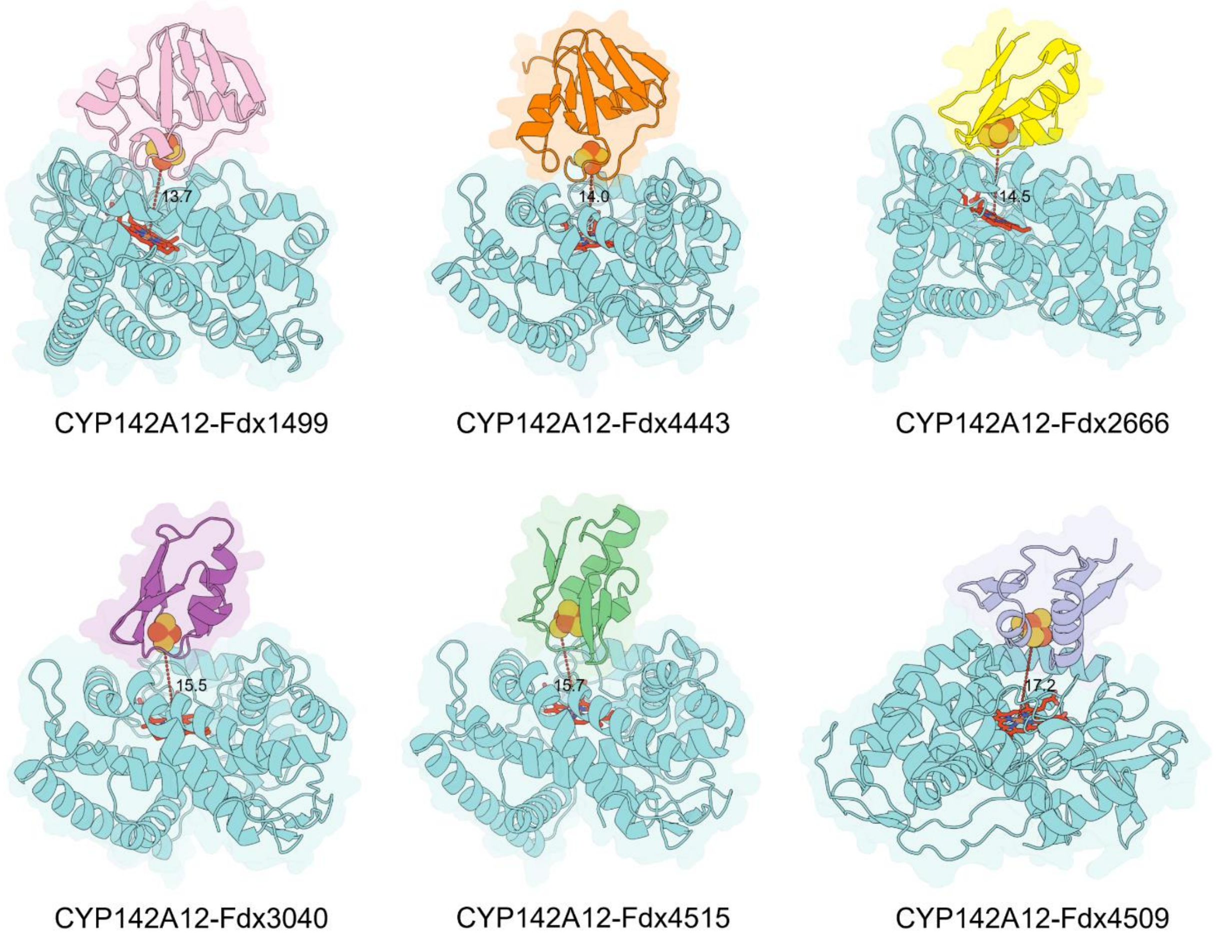

Figure S22. Fdx-P450 docking models. The structures of P450-Fdx complexes are shown as cartoon with different colors. Heme is shown as sticks in red. The Fe_2S_2 cluster and Fe_3S_4 cluster are shown as spheres. The distances (Å) between the iron–sulfur cluster and heme-iron are indicated by dashed red lines.

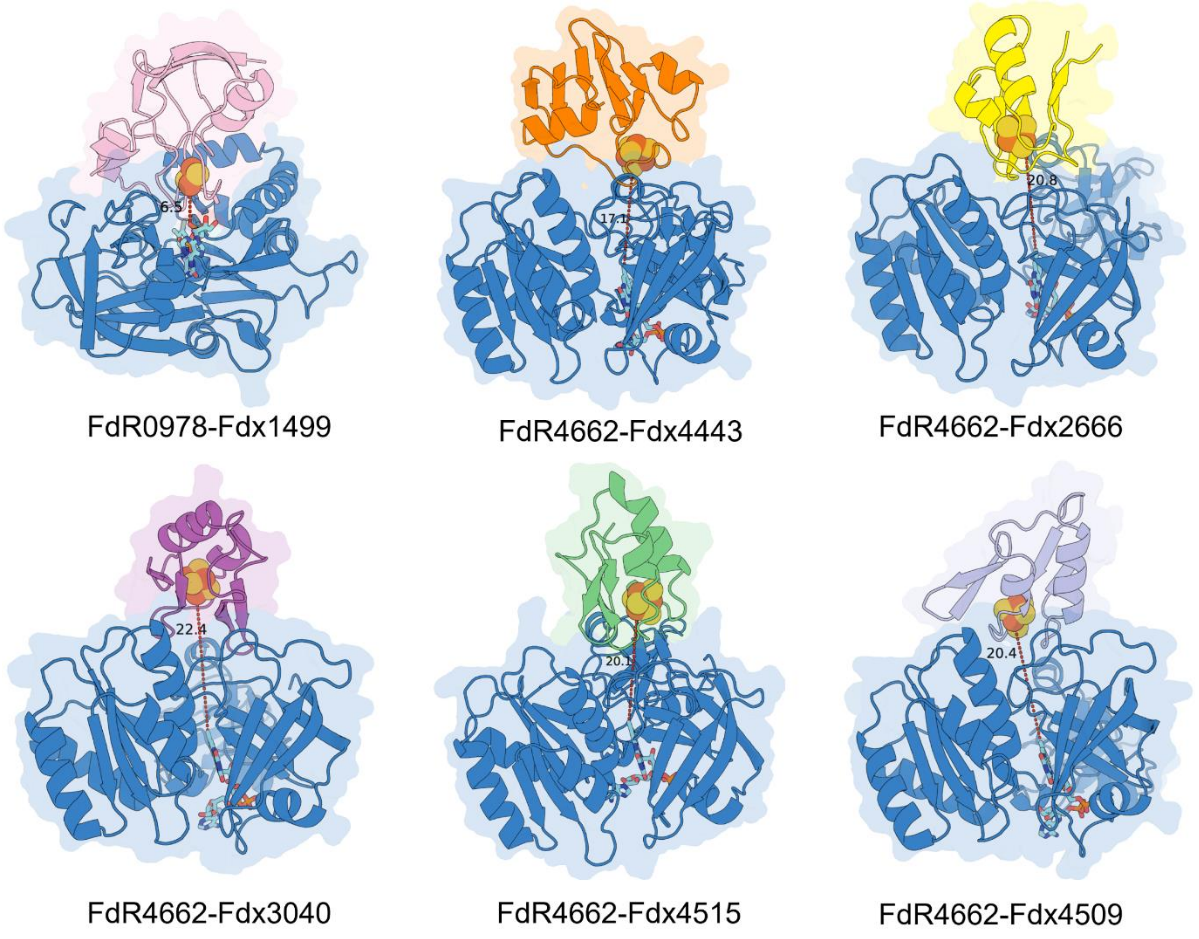

Figure S23. Fdx-FdR docking models. The structures of FdR-Fdx complexes are shown as cartoon with different colors. FAD is shown as sticks in cyan. The Fe_2S_2 cluster and Fe_3S_4 cluster shown as spheres. The shortest FAD-Fe_2S_2 and FAD- Fe_3S_4 distances (Å) are indicated by dashed red lines.

Supplementary Tables

Table S1. Characteristics of P450, Fdx and FdR genes from *M. neoaurum* ZC-1.

Gene name	Prorein ID	GeneBank number	Molecular weighet (MD)	Number of amio acids
CYP51B1	BN1047_03078	CDQ45189.1	51.66 KDa	452
CYP102U1	BN1047_01030	CDQ43167.1	113.58 KDa	1049
CYP105Q9	BN1047_04463	CDQ46554.1	45.37 KDa	405
CYP105S17	BN1047_00441	CDQ42586.1	43.57 KDa	390
CYP108B24	BN1047_04787	CDQ46873.1	47.27 KDa	421
CYP123A28	BN1047_03080	CDQ45191.1	45.02 KDa	400
CYP123B2	BN1047_02775	CDQ44895.1	43.84 KDa	398
CYP124A1	BN1047_02480	CDQ44600.1	48.07 KDa	432
CYP124P3	BN1047_02589	CDQ44709.1	45.36 KDa	407
CYP125A76	BN1047_03088	CDQ45199.1	45.87 KDa	411
CYP125A77	BN1047_02411	CDQ44531.1	47.01 KDa	424
CYP125A78	BN1047_04993	CDQ47075.1	46.08 KDa	417
CYP126A6	BN1047_03095	CDQ45206.1	44.66 KDa	405
CYP130A25	BN1047_03765	CDQ45865.1	44.64 KDa	406
CYP135B9	BN1047_02490	CDQ44610.1	48.81 KDa	444
CYP136A1	BN1047_00684	CDQ42826.1	55.81 KDa	493
CYP138A10	BN1047_01525	CDQ43654.1	49.48 KDa	440
CYP136B10	BN1047_00373	CDQ42520.1	53.55 KDa	479
CYP140A10	BN1047_01257	CDQ43392.1	48.24 KDa	440
CYP145C5	BN1047_00188	CDQ42336.1	43.83 KDa	396
CYP142A12	BN1047_03021	CDQ47378.1	45.37 KDa	407
CYP144A5	BN1047_03600	CDQ45700.1	44.20 KDa	404
CYP150A3	BN1047_04495	CDQ46586.1	47.09 KDa	425
CYP150A25	BN1047_04547	CDQ46638.1	47.97 KDa	425
CYP164A10	BN1047_02378	CDQ44498.1	45.24 KDa	416
CYP188A9	BN1047_04525	CDQ46616.1	51.62 KDa	455
CYP189A1	BN1047_04490	CDQ46581.1	44.37 KDa	392
CYP190A1	BN1047_04510	CDQ46601.1	44.98 KDa	400
CYP268A3	BN1047_04682	CDQ46768.1	46.39 KDa	421
CYP268J1	BN1047_02637	CDQ44757.1	50.02 KDa	451

CYP268F1	BN1047_02669	CDQ44789.1	46.55 KDa	418
CYP268F2	BN1047_02663	CDQ44783.1	48.74 KDa	437
Fdx0440	BN1047_00440	CDQ42585.1	4.02 KDa	63
Fdx1955	BN1047_01955	CDQ44079.1	6.29 KDa	75
Fdx2638	BN1047_02638	CDQ44758.1	4.26 KDa	67
Fdx2666	BN1047_02666	CDQ44786.1	6.31 KDa	61
Fdx4526	BN1047_04526	CDQ46617.1	4.87 KDa	63
Fdx4515	BN1047_04515	CDQ46606.1	11.00 KDa	97
Fdx4509	BN1047_04509"	CDQ46600.1	7.28 KDa	64
Fdx4496	BN1047_04496	CDQ46587.1	6.63 KDa	62
Fdx4462	BN1047_04462	CDQ46553.1	4.76 KDa	64
Fdx4443	BN1047_04443	CDQ46534.1	10.18 KDa	98
Fdx3077	BN1047_03077	CDQ45188.1	7.66 KDa	68
Fdx3040	BN1047_03040	CDQ45152.1	6.88 KDa	63
Fdx1858	BN1047_01858	CDQ43982.1	12.27 KDa	117
Fdx3698	BN1047_03698	CDQ45798.1	11.61 KDa	106
FdR1588	BN1047_01588	CDQ43717.1	47.97 KDa	446
FdR1277	BN1047_01277	CDQ43411.1	53.45 KDa	567
FdR4258	BN1047_04258	CDQ46351.1	59.29 KDa	556
FdR4150	BN1047_04150	CDQ46245.1	38.28 KDa	355
FdR4041	BN1047_04041	CDQ46137.1	41.81 KDa	388
FdR3863	BN1047_03863	CDQ45960.1	29.18 KDa	256
FdR3862	BN1047_03862	CDQ45959.1	17.43 KDa	168
FdR0600	BN1047_00600	CDQ42743.1	39.72 KDa	371
FdR4662	BN1047_04662	CDQ46748.1	32.97 KDa	311
FdR3233	BN1047_03233	CDQ45338.1	54.84 KDa	549
FdR0342	BN1047_00342	CDQ42489.1	54.74 KDa	525

Table S2. Expression vectors and heterologous expression profiles of P450, Fdx, and FdR genes from *M. neoaurum* ZC-1.

Protein name	Host System	Vector	Fusion Tag	Solubility
CYP51B1	*E. coli* BL21(DE3)	pMAL-c5E	N-terminal MBP-tag	Soluble
CYP102U1	*E. coli* BL21(DE3)	pMAL-c5E	N-terminal MBP-tag	Insoluble
CYP105Q9	*E. coli* BL21(DE3)	pMAL-c5E	N-terminal MBP-tag	Soluble
CYP105S17	*E. coli* BL21(DE3)	pET22b	N-terminal His-tag	Soluble
CYP108B24	*E. coli* BL21(DE3)	pMAL-c5E	N-terminal MBP-tag	Soluble
CYP123A28	*E. coli* BL21(DE3)	pMAL-c5E	N-terminal MBP-tag	Soluble
CYP123B2	*E. coli* BL21(DE3)	pMAL-c5E	N-terminal MBP-tag	Soluble
CYP124A1	*E. coli* BL21(DE3)	pET32a	N-terminal His-tag	Soluble
CYP124P3	*E. coli* BL21(DE3)	pET32a	N-terminal His-tag	Insoluble
CYP125A76	*E. coli* BL21(DE3)	pET32a	N-terminal His-tag	Soluble
CYP125A77	*E. coli* BL21(DE3)	pET32a	N-terminal His-tag	Soluble
CYP125A78	*E. coli* BL21(DE3)	pET22b	N-terminal His-tag	Soluble
CYP126A6	*E. coli* BL21(DE3)	pMAL-c5E	N-terminal MBP-tag	Soluble
CYP130A25	*E. coli* BL21(DE3)	pMAL-c5E	N-terminal MBP-tag	Soluble
CYP135B9	*E. coli* BL21(DE3)	pMAL-c5E	N-terminal MBP-tag	Insoluble
CYP136A1	*E. coli* BL21(DE3)	pMAL-c5E	N-terminal MBP-tag	Insoluble
CYP138A10	*E. coli* BL21(DE3)	pMAL-c5E	N-terminal MBP-tag	Insoluble
CYP136B10	*E. coli* BL21(DE3)	pMAL-c5E	N-terminal MBP-tag	Insoluble
CYP140A10	*E. coli* BL21(DE3)	pMAL-c5E	N-terminal MBP-tag	Soluble
CYP145C5	*E. coli* BL21(DE3)	pMAL-c5E	N-terminal MBP-tag	Insoluble
CYP142A12	*E. coli* BL21(DE3)	pET32a	N-terminal His-tag	Soluble
CYP144A5	*E. coli* BL21(DE3)	pMAL-c5E	N-terminal MBP-tag	Soluble
CYP150A3	*E. coli* BL21(DE3)	pMAL-c5E	N-terminal MBP-tag	Soluble
CYP150A25	*E. coli* BL21(DE3)	pMAL-c5E	N-terminal MBP-tag	Soluble
CYP164A10	*E. coli* BL21(DE3)	pMAL-c5E	N-terminal MBP-tag	Soluble
CYP188A9	*E. coli* BL21(DE3)	pMAL-c5E	N-terminal MBP-tag	Insoluble
CYP189A1	*E. coli* BL21(DE3)	pMAL-c5E	N-terminal MBP-tag	Soluble
CYP190A1	*E. coli* BL21(DE3)	pMAL-c5E	N-terminal MBP-tag	Soluble
CYP268A3	*E. coli* BL21(DE3)	pMAL-c5E	N-terminal MBP-tag	Soluble
CYP268J1	*E. coli* BL21(DE3)	pMAL-c5E	N-terminal MBP-tag	Soluble

CYP268F1	*E. coli* BL21(DE3)	pMAL-c5E	N-terminal MBP-tag	Soluble
CYP268F2	*E. coli* BL21(DE3)	pET22b	N-terminal His-tag	Soluble
Fdx0440	*E. coli* BL21(DE3)	pMAL-c5E	N-terminal MBP-tag	Insoluble
Fdx1955	*E. coli* BL21(DE3)	pMAL-c5E	N-terminal MBP-tag	Soluble
Fdx2638	*E. coli* BL21(DE3)	pMAL-c5E	N-terminal MBP-tag	Insoluble
Fdx2666	*E. coli* BL21(DE3)	pMAL-c5E	N-terminal MBP-tag	Soluble
Fdx4526	*E. coli* BL21(DE3)	pMAL-c5E	N-terminal MBP-tag	Soluble
Fdx4515	*E. coli* BL21(DE3)	pMAL-c5E	N-terminal MBP-tag	Soluble
Fdx4509	*E. coli* BL21(DE3)	pMAL-c5E	N-terminal MBP-tag	Soluble
Fdx4496	*E. coli* BL21(DE3)	pMAL-c5E	N-terminal MBP-tag	Soluble
Fdx4462	*E. coli* BL21(DE3)	pMAL-c5E	N-terminal MBP-tag	Soluble
Fdx4443	*E. coli* BL21(DE3)	pMAL-c5E	N-terminal MBP-tag	Soluble
Fdx3077	*E. coli* BL21(DE3)	pMAL-c5E	N-terminal MBP-tag	Soluble
Fdx3040	*E. coli* BL21(DE3)	pMAL-c5E	N-terminal MBP-tag	Soluble
Fdx1858	*E. coli* BL21(DE3)	pMAL-c5E	N-terminal MBP-tag	Soluble
Fdx3698	*E. coli* BL21(DE3)	pMAL-c5E	N-terminal MBP-tag	Soluble
FdR1588	*E. coli* BL21(DE3)	pMAL-c5E	N-terminal MBP-tag	Insoluble
FdR1277	*E. coli* BL21(DE3)	pMAL-c5E	N-terminal MBP-tag	Soluble
FdR4258	*E. coli* BL21(DE3)	pMAL-c5E	N-terminal MBP-tag	Soluble
FdR4150	*E. coli* BL21(DE3)	pMAL-c5E	N-terminal MBP-tag	Soluble
FdR4041	*E. coli* BL21(DE3)	pMAL-c5E	N-terminal MBP-tag	Soluble
FdR3863	*E. coli* BL21(DE3)	pMAL-c5E	N-terminal MBP-tag	Soluble
FdR3862	*E. coli* BL21(DE3)	pMAL-c5E	N-terminal MBP-tag	Soluble
FdR0600	*E. coli* BL21(DE3)	pMAL-c5E	N-terminal MBP-tag	Soluble
FdR4662	*E. coli* BL21(DE3)	pMAL-c5E	N-terminal MBP-tag	Soluble
FdR3233	*E. coli* BL21(DE3)	pMAL-c5E	N-terminal MBP-tag	Soluble
FdR0342	*E. coli* BL21(DE3)	pMAL-c5E	N-terminal MBP-tag	Soluble

Table S3. Classification of Fdxs in *M. neoaurum* ZC-1.

**Gene name**	**Classification**	**Fe-S cluster binding motif**
Fdx0440	Fe_3S_4	CX_5CX_nCP
Fdx1955	Fe_2S_2	$CX_5CX_2CX_{15}CP$
Fdx2638	Fe_3S_4	CX_5CX_nCP
Fdx2666	Fe_3S_4	$CX_5CX_nC_P$
Fdx4526	Fe_2S_2	$CX_5CX_2CX_{35-37}C$
Fdx4515	Fe_3S_4	$CX_5CX_nC_P$
Fdx4509	Fe_3S_4	$CX_5CX_nC_P$
Fdx4496	Fe_2S_2	$CX_5CX_3CPX_{34}CP$
Fdx4462	Fe_2S_2	$CX_5CX_2CX_{35-37}C$
Fdx4443	Fe_3S_4	CX_5CX_nCP
Fdx3077	Fe_3S_4	CX_5CX_nCP
Fdx3040	Fe_3S_4	CX_5CX_nCP
Fdx1858	Fe_7S_8	CX_7C and CX_2CGXCX_3CR
Fdx3698	Fe_7S_8	CX_7C and CX_2CGXCX_3CP

Table S4. PCR primers used in this study.

Primer name	Primer sequence (5’→3’)
CYP108B24-F	AATTAATTCGGATCCGAATTCGATGAGCACTCCGAC
CYP108B24-R	GTGGTGGTGGTGGTGGTGCTCGAGGCGCAGCGAGTACC
CYP105S17-F	AATTAATTCGGATCCGAATTCGATGCTGCCACCGGTCCACA
CYP105S17-R	GTGGTGGTGGTGGTGGTGCTCGAGCCACCGCACCGATAGCG
CYP140A10-F	TTAATTCGGATCCGAATTCGATGGCGACAATGCGTGAACGTATCCA
CYP140A10-R	GTGGTGGTGGTGGTGGTGCTCGAGCGACGGTAGCGCGGCGC
CYP138A10-F	AATTAATTCGGATCCGAATTCGATGAGCGAAGCGACCGTCGAACG
CYP138A10-R	GTGGTGGTGGTGGTGGTGCTCGAGCCGGGCGTGTAGCACCACGCG
CYP142A12-F	AATTAATTCGGATCCGAATTCGATGACAGGGGTTCTGACCG
CYP142A12-R	GTGGTGGTGGTGGTGGTGCTCGAGGCCGCGCACCTTCGCCG
CYP51B1-F	AATTAATTCGGATCCGAATTCGATGGCTGATCTGAAGGAAGTAGA
CYP51B1-R	GTGGTGGTGGTGGTGGTGCTCGAGGTCCCGGACTCGCCTGC
CYP123A28-F	GAATTAATTCGGATCCGAATTCGATGAGCTCTCCCACCGTC
CYP123A28-R	GTGGTGGTGGTGGTGGTGCTCGAGACGGAGGGTGACGGTCATCGG
CYP125A76-F	AATTAATTCGGATCCGAATTCGATGACGACGATGGATGCGTGTC
CYP125A76-R	GTGGTGGTGGTGGTGGTGCTCGAGGGAGTAGGAAACCTGCAGTTC
CYP126A6-F	AATTAATTCGGATCCGAATTCGACACTGGCCGACCTCACCGATCT
CYP126A6-R	GTGGTGGTGGTGGTGCTCGAGGCCGAACTCGACGAACATGTGCC
CYP144A5-F	AATTAATTCGGATCCGAATTCGATGACCTTGTCAGCGGATGTG
CYP144A5-R	GTGGTGGTGGTGGTGGTGCTCGAGGCGCACCGCCAGGGTCAGGTG
CYP130A25-F	AATTAATTCGGATCCGAATTCGATGTCTCACGATGCGGTCTTC
CYP130A25-R	GTGGTGGTGGTGGTGGTGCTCGAGTCGGCATACGGTGAACGGTACC
CYP125A77-F	AATTAATTCGGATCCGAATTCGATGACCGACACCGCCCACG
CYP125A77-R	GTGGTGGTGGTGGTGGTGCTCGAGTTGGTGCACAGCCTGTTTCGAC
CYP164A10-F	AATTAATTCGGATCCGAATTCGATGACCTCCGTGTCCACCC
CYP164A10-R	GTGGTGGTGGTGGTGGTGCTCGAGCACGGTCACCGGCAACGACA
CYP105Q9-F	AATTAATTCGGATCCGAATTCGATGCTGACCGAGACCGAAG
CYP105Q9-R	GTGGTGGTGGTGGTGGTGCTCGAGCCAGGTCACCGGGAGTTCG
CYP189A1-F	AATTAATTCGGATCCGAATTCGATGAGCGCACCGACCGAGGCCGA
CYP189A1-R	GTGGTGGTGGTGGTGGTGCTCGAGGACCAGTGTCGGCATCGCCGAC
CYP150A3-F	AATTAATTCGGATCCGAATTCGATGTCTCACGATGCGGTCTTC
CYP150A3-R	GTGGTGGTGGTGGTGGTGCTCGAGTCGGCATACGGTGAACGGTACC

CYP190A1-F	AATTAATTCGGATCCGAATTCGATGACCACCACCGACCTCGCG
CYP190A1-R	GTGGTGGTGGTGGTGGTGCTCGAGTGGCAGTCTCCGTACCG
CYP188A9-F	AATTAATTCGGATCCGAATTCGATGACCATCGATGACATCTCGGCCGACG
CYP188A9-R	GTGGTGGTGGTGGTGGTGCTCGAGCTTCCAGTCGATGACGG
CYP150A25-F	AATTAATTCGGATCCGAATTCGATGAGCGAATACGCGACGCTG
CYP150A25-R	GTGGTGGTGGTGGTGGTGCTCGAGGCTCAGCGGGGTGTAGTTG
CYP268A3-F	AATTAATTCGGATCCGAATTCGACAGTGTCACCGATCACAACG
CYP268A3-R	GTGGTGGTGGTGGTGGTGCTCGAGGAAGCTGCAGGGCAGCGAAC
CYP102U1-F	AATTAATTCGGATCCGAATTCGATGACCGAGTTCGCGCCGTCG
CYP102U1-R	GTGGTGGTGGTGGTGGTGCTCGAGCCCAGCCCACACATCCTCGACGTAAT
CYP136B10-F	AATTAATTCGGATCCGAATTCGACAGTGTCACCGATCACAACG
CYP136B10-R	GTGGTGGTGGTGGTGGTGCTCGAGCCCAGCCCACACATCCTCGACGTAAT
CYP136A1-F	ATTAATTCGGATCCGAATTCGGTGACGACCGTCCTGCCCGCC
CYP136A1-R	GTGGTGGTGGTGGTGCTCGAGGTCCAGTCGGCGCATCTCGATC
CYP145C-F	AATTAATTCGGATCCGAATTCGATGGCGACAACCATCA
CYP145C-R	GTGGTGGTGGTGGTGGTGCTCGAGGAGTGGACGCAGGA
CYP124A1-F	ATTAATTCGGATCCGAATTCGATGTCGATCGCGGTCCGTCGCAA
CYP124A1-R	GTGGTGGTGGTGGTGGTGCTCGAGGCCGATGCCTTGAGCCCCG
CYP135B9-F	ATTAATTCGGATCCGAATTCGATGACCGAATTGGCGCTGCCGCCC
CYP135B9-R	GTGGTGGTGGTGGTGGTGCTCGAGCTGCGCTGACGCCGCCGCAT
CYP124P3-F	ATTAATTCGGATCCGAATTCGATGCTCGGTTCGATCATTG
CYP124P3-R	GTGGTGGTGGTGGTGGTGCTCGAGTTCGATGGAGCAGGGGAGCGCTT
CYP268J1-F	AATTAATTCGGATCCGAATTCGATGACCGAGTTCGCGCCGTCG
CYP268J1-R	GTGGTGGTGGTGGTGGTGCTCGAGCCAGGTCACCGGGAGTTCG
CYP268F2-F	AATTAATTCGGATCCGAATTCGATGACCAGCAGCCCG
CYP268F2-R	GTGGTGGTGGTGGTGGTGCTCGAGGTGTCCGACCGGAC
CYP268F1-F	ATCGGAATTAATTCGGATCCGAATTCGATGAGTGCGCCCACTGCGAC
CYP268F1-R	GTGGTGGTGGTGGTGGTGCTCGAGATCCAGGCTGTAGGAAAGGGATTTG
CYP123B2-F	AATTAATTCGGATCCGAATTCGGTCTCGGTGCAGATTCGT
CYP123B2-R	GTGGTGGTGGTGGTGGTGCTCGAGGACCGGTGTGGTCCA
CYP125A78-F	AATTAATTCGGATCCGAATTCGATGCCCAGCCCCAA
CYP125A78-R	GTGGTGGTGGTGGTGGTGCTCGAGGCTCGAGGCGCCG
FdR1588-F	TGCCGCGCGGCAGAAGCTTGGTGGCGGTCATCGGAGCCGG
FdR1588-R	CAGTGGTGGTGGTGGTGGTGCTCGAGGGCGCGCCCTGCCG

FdR1277-F	CGGCAGAAGCTTGGTGTCCATCACCATCCAACAGCCCGCC
FdR1277-R	CAGTGGTGGTGGTGGTGGTGCTCGAGGGCGCGCCCTGCCG
FdR4258-F	ACTGGTGCCGCGCGGCAGAAGCTTGATGGCATACGTGATCACCCGGTC
FdR4258-R	TGGTGGTGGTGGTGCTCGAGTGAGTTTCTCCTAAATTGATCGACCGACTG
FdR4150-F	CGGCAGAAGCTTGGTGTCCATCACCATCCAACAGCCCGCC
FdR4150-R	TGGTGGTGGTGGTGCTCGAGGAGGTCGATGGCGACATCGC
FdR4041-F	CTGGTGCCGCGCGGCAGAAGCTTGGTGGCCAAGAATGAAA
FdR4041-R	GTGGTGGTGGTGGTGGTGCTCGAGTGCGTCCAACGTGCAG
FdR3863-F	CTGGTGCCGCGCGGCAGAAGCTTGGTGCAGGAGGTGATCGTGGTGGG
FdR3863-R	TCAGTGGTGGTGGTGGTGGTGCTCGAGGCCGTGCGCTATCCCCAGCA
FdR3862-F	TGGTGCCGCGCGGCAGAAGCTTGGTGGCGATCGTCGGCTC
FdR3862-R	GTGGTGGTGGTGGTGGTGCTCGAGTGCGTCCAACGTGCAG
FdR0600-F	GCGCGGCAGAAGCTTGGTGTCGCGGCTGCTGCGACCGTAC
FdR0600-R	GTGGTGGTGGTGGTGCTCGAGCTACAGGTCCAGCGTCAGCC
FdR4662-F	CTGGTGCCGCGCGGCAGAAGCTTGATGAGAACCCTCAAGC
FdR4662-R	TGGTGGTGGTGGTGGTGCTCGAGTCACAACGCCACCTCCAGTGT
FdR3233-F	CTGGTGCCGCGCGGCAGAAGCTTGATGCTCTACATCGACCCGCT
FdR3233-R	TGGTGGTGGTGGTGCTCGAGTCAGCGCCGCAGCCCCGCGAGCAG
FdR0342-F	CTGGTGCCGCGCGGCAGAAGCTTGGTGCCGGTCTGCCCGG
FdR0342-R	GTGGTGGTGGTGGTGGTGCTCGAGCGATCCCGCCGTCACCG
Fdx0440-F	GCCGCGCGGCAGAAGCTTGGTGATGACCGCC
Fdx0440-R	GTGGTGGTGGTGGTGGTGCTCGAGCTCGACCAC
Fdx1955-F	GCGGCAGGAGCTTGATGTTCGTCTGCCTGTGCACCGGC
Fdx1955-R	TGGTGGTGGTGCTCGAGTCAACCCGCGGCGT
Fdx2638-F	GCTTGGTGCTCATCGACATCGACAGGTGCTCGGGT
Fdx2638-R	TGGTGGTGGTGGTGGTGCTCGAGTCAGGCGGTCTG
Fdx2666-F	GAAGCTTGATGGGAGTACGGGTCGACGGCTCCAGG
Fdx2666-R	GGTGGTGGTGGTGGTGCTCGAGCTAGTCGTCGAAC
Fdx4526-F	GAAGCTTGATGATCGCGCCCGACGCGTTCGAACTC
Fdx4526-R	GTGGTGGTGCTCGAGTCATTCGATGATGATGGCCT
Fdx4515-F	GGCAGAAGCTTGATGCGGATCAAACTCGACCGGAC
Fdx4515-R	GGTGGTGGTGGTGGTGCTCGAGTCATCGTGTCATA
Fdx4509-F	CAGAAGCTTGATGAGTCGCGTCGAAGTCGACTTCG
Fdx4509-R	GTGGTGGTGGTGGTGCTCGAGCTCGACGATCGTGA

Fdx4496-F	GGCAGAAGCTTGATGAAGGTCGAAGTGGACGAGGA
Fdx4496-R	GTGGTGGTGGTGGTGGTGCTCGAGGAGCTTGCGGA
Fdx4462-F	CAGAAGCTTGATGGACGTGTTCGACCAGCGTGACG
Fdx4462-R	GTGGTGGTGGTGCTCGAGGTCCTGGATGTCGATGG
Fdx4443-F	ATGACGGCCGAACCCGTGCCTGCCGACG
Fdx4443-R	GGTGGTGGTGGTGCTCGAGGTCGTCGTAGTTCACC
Fdx3077-F	ACGTTGATGGGTTGCTATCGCATAGAACTCG
Fdx3077-R	GTGGTGGTGGTGGTGCTCGAGGTCTTTTTCGATTAT
Fdx3040-F	AGCTTGATGCACGTAGAAGTCGACCGTGACCGCTG
Fdx3040-R	TGGTGGTGGTGGTGGTGCTCGAGGTCTCTACGGAT
Fdx1858 -F	GCGCGGCAGAAGCTTGATGACCTACGTGATCGGAT
Fdx1858 -R	TGGTGGTGGTGGTGCTCGAGGTGCGCGGGCACGTC
Fdx3698-F	GCGGCAGAAGCTTGGTGACGTACGTCATTGCCGAGCC
Fdx3698-R	GTGGTGGTGGTGGTGGTGCTCGAGCTCGCCCTTGG

Table S5. Electron transfer rates for *cyt c* reduction (μM·s⁻¹·μM⁻¹) by different redox partner combinations (10 FdRs and 12Fdxs).

	**FdR1277**	**FdR4258**	**FdR4150**	**FdR4041**	**FdR3863**	**FdR3862**	**FdR0600**	**FdR4662**	**FdR3233**	**FdR0342**
**no Fdx**	0.09±0.01	0.03±0.03	0.10±0.04	0.04±0.01	0.07±0.01	0.05±0.00	0.10±0.01	0.10±0.01	0.09±0.03	0.07±0.02
**Fdx1955**	0.11±0.00	0.11±0.00	0.11±0.00	0.07±0.01	0.11±0.01	0.10±0.00	0.09±0.01	0.17±0.01	0.09±0.00	0.10±0.01
**Fdx2666**	0.11±0.02	0.10±0.01	0.14±0.00	0.06±0.02	0.14±0.00	0.08±0.00	0.15±0.02	0.25±0.02	0.16±0.00	0.08±0.01
**Fdx4526**	0.18±0.02	0.10±0.03	0.08±0.04	0.08±0.02	0.13±0.01	0.11±0.01	0.13±0.02	0.18±0.01	0.12±0.02	0.07±0.01
**Fdx4515**	0.12±0.01	0.10±0.01	0.12±0.01	0.06±0.01	0.11±0.02	0.09±0.01	0.16±0.02	0.24±0.00	0.09±0.01	0.12±0.01
**Fdx4509**	0.11±0.01	0.10±0.01	0.11±0.01	0.07±0.00	0.12±0.00	0.10±0.04	0.09±0.01	0.12±0.02	0.10±0.01	0.08±0.01
**Fdx4496**	0.18±0.02	0.13±0.02	0.10±0.01	0.13±0.01	0.18±0.03	0.15±0.01	0.10±0.01	0.19±0.01	0.14±0.00	0.08±0.00
**Fdx4462**	0.16±0.02	0.03±0.02	0.12±0.01	0.07±0.02	0.15±0.02	0.12±0.02	0.17±0.02	0.16±0.02	0.12±0.02	0.16±0.02
**Fdx4443**	0.14±0.01	0.16±0.01	0.11±0.02	0.08±0.01	0.15±0.01	0.16±0.04	0.15±0.01	0.37±0.04	0.16±0.01	0.19±0.00
**Fdx3077**	0.10±0.02	0.09±0.01	0.05±0.01	0.03±0.01	0.15±0.03	0.08±0.04	0.11±0.04	0.18±0.01	0.19±0.01	0.14±0.02
**Fdx3040**	0.14±0.01	0.10±0.01	0.14±0.02	0.10±0.01	0.11±0.02	0.07±0.00	0.15±0.01	0.31±0.02	0.14±0.01	0.16±0.02
**Fdx1858**	0.11±0.03	0.11±0.01	0.13±0.03	0.13±0.02	0.16±0.01	0.13±0.02	0.12±0.01	0.18±0.02	0.20±0.01	0.09±0.02
**Fdx3698**	0.07±0.01	0.09±0.03	0.11±0.02	0.11±0.00	0.15±0.04	0.06±0.01	0.13±0.03	0.10±0.02	0.13±0.02	0.06±0.02

Table S6. Conversion ratios (%) of 7-dehydrocholesterol by CYP125A77 when supported by 120 different redox partners pairs (10 FdRs × 12 Fdxs). N.D., not detected.

	**FdR1277**	**FdR4258**	**FdR4150**	**FdR4041**	**FdR3863**	**FdR3862**	**FdR0600**	**FdR4662**	**FdR3233**	**FdR0342**
**Fdx1955**	N.D.	N.D.	N.D.	N.D.	N.D.	N.D.	N.D.	N.D.	N.D.	N.D.
**Fdx2666**	4.0±1.5	10.0±2.1	1.9±0.7	4.5±0.7	5.8±1.1	4.5±1.6	1.0±0.3	47.3±2.5	10.0±1.2	1.9±1.3
**Fdx4526**	N.D.	N.D.	N.D.	N.D.	N.D.	N.D.	N.D.	N.D.	N.D.	N.D.
**Fdx4515**	12.5±1.4	2.3±0.8	3.3±0.6	1.4±0.7	5.3±0.9	0.8±0.1	1.0±0.3	34.0±2.5	2.6±0.5	1.9±0.9
**Fdx4509**	N.D.	N.D.	N.D.	N.D.	N.D.	N.D.	N.D.	N.D.	N.D.	N.D.
**Fdx4496**	N.D.	N.D.	N.D.	N.D.	N.D.	N.D.	N.D.	N.D.	N.D.	N.D.
**Fdx4462**	N.D.	N.D.	N.D.	N.D.	N.D.	N.D.	N.D.	N.D.	N.D.	N.D.
**Fdx4443**	12.5±1.5	8.4±1.7	7.5±1.0	11.0±1.1	7.0±0.5	8.0±1.1	3.5±1.0	55.3±1.6	15.0±1.2	12.0±1.4
**Fdx3077**	N.D.	N.D.	N.D.	N.D.	N.D.	N.D.	N.D.	N.D.	N.D.	N.D.
**Fdx3040**	16.5±1.2	19.0±1.2	14.0±1.4	3.5±0.8	5.5±1.1	7.7±1.4	3.0±1.1	49.7±3.1	1.7±0.6	19.0±1.7
**Fdx1858**	N.D.	N.D.	N.D.	N.D.	N.D.	N.D.	N.D.	N.D.	N.D.	N.D.
**Fdx3698**	N.D.	N.D.	N.D.	N.D.	N.D.	N.D.	N.D.	N.D.	N.D.	N.D.

References

1. Li, S., Podust, L. M., and Sherman, D. H. (2007) Engineering and analysis of a self-sufficient biosynthetic cytochrome p450 PikC fused to the RhFRED reductase domain *J. Am. Chem. Soc.* **129**, 12940-12941
2. Omura, T., and Sato, R. (1964) The carbon monoxide-binding pigment of liver microsomes: II. Solubilization, purification, and properties *J. Biol. Chem.* **239**, 2379-2385
3. Notredame, C. D., Higgins, D. G., and Heringa, J. (2000) T-coffee: a novel method for fast and accurate multiple sequence alignment *J. Mol. Biol.* **302**, 205-217
4. Robert, X., and Gouet, P. (2014) Deciphering key features in protein structures with the new ENDscript server *Nucleic. Acids. Res.* **42**, W320-W324
5. Sezutsu, H., Le Goff, G. L., and Feyereisen, R. (2013) Origins of p450 diversity *Philos. Trans. R. Soc. B-Biol. Sci.* **368**, 20120428
